# Cytoprotective effects and antioxidant activities of acteoside and various extracts of *Clerodendrum cyrtophyllum* Turcz leaves against *t*-BHP induced oxidative damage

**DOI:** 10.1038/s41598-022-17038-w

**Published:** 2022-07-25

**Authors:** Junjie Zhu, Gang Li, Jing Zhou, Zhiyong Xu, Jing Xu

**Affiliations:** 1 School of Chemical Engineering and Technology, Hainan University, Haikou, 570228 People’s Republic of China; 2 School of Life and Pharmaceutical Sciences, Hainan University, Haikou, 570228 People’s Republic of China

**Keywords:** Biochemistry, Cell biology, Drug discovery, Health care

## Abstract

This study evaluates the antioxidant potential and cytoprotective effects of ethanolic crude extract from *Clerodendrum cyrtophyllum* leaves (ECE) and five derived fractions (namely, petroleum ether fraction (PEF), dichloromethane fraction (DMF), ethyl acetate fraction (EAF), *n*-butyl alcohol fraction (BAF) and the remaining fraction (RF)), as well as acteoside (Ac, a major phenolic component in EAF) on oxidative damage caused by *tert*-butyl hydroperoxide (*t*-BHP) in HepG2 cells. MTT assay results showed that ECE, EAF, BAF, RF and Ac increased the viability of *t*-BHP-damaged cells in a dose-dependent manner, while EAF significantly promoted cell viability. EAF, BAF, RF, or Ac reduced the levels of lactate dehydrogenase (LDH) leakage, malondialdehyde (MDA), and reactive oxygen species (ROS). Additionally, glutathione (GSH) levels and the activities of superoxide dismutase (SOD) and catalase (CAT) increased. Western blot analysis further indicated that EAF, BAF, RF, or Ac up-regulated pro-caspase-3 and reduced cleaved caspase-3 during *t*-BHP-induced oxidative stress. Flow cytometry analysis and fluorescence micrographs showed that Ac could inhibit apoptosis.

## Introduction

Reactive oxygen species (ROS), such as superoxide anion radical (O_2_^−^), hydrogen peroxide (H_2_O_2_), and hydroxyl radical (OH**·**), are highly reactive non-specific molecules and by-products of biological metabolism^1^. In humans, the disturbed ROS production-scavenging system is a key event in the progression of oxidative damage of biological molecules related to degenerative or pathological processes, such as aging, cancer, atherosclerosis, gastric ulcer, and other conditions^2^. Accumulation of ROS can trigger and exacerbate the peroxidation of cell membrane lipids and induce oxidative stress. As a result, the release of LDH and the production of MDA increase, which are important biomarkers for lipid peroxidation^3^. Almost all organisms are well protected against oxidative damage by endogenous ROS scavenging enzymes like superoxide dismutase (SOD), catalase (CAT), glutathione peroxidase (GSHPx), and non-enzymatic endogenous antioxidant substances like reduced glutathione (GSH), which can efficiently absorb ROS and prevent them from attacking other essential proteins. However, ROS scavenging systems cannot prevent damage entirely; thus, exogenous antioxidants are vitally important in maintaining health^4^.

Synthetic antioxidants, such as butylated hydroxyanisole (BHA), butylated hydroxytoluene (BHT), and *tert*-butyl hydroquinone (TBHQ), have been widely used in food and pharmaceutical products, but their use is often accompanied by toxic and carcinogenic effects, as well as abnormal effects on enzyme systems^5^. Safer, natural antioxidants are being extensively studied for their capacity to scavenge and neutralize free radicals to reduce oxidative stress and to restore cellular homeostasis^6^. Many of these natural antioxidants are derived from plant origins, such as Chinese medicinal herbals^7^.

**Figure 1:**
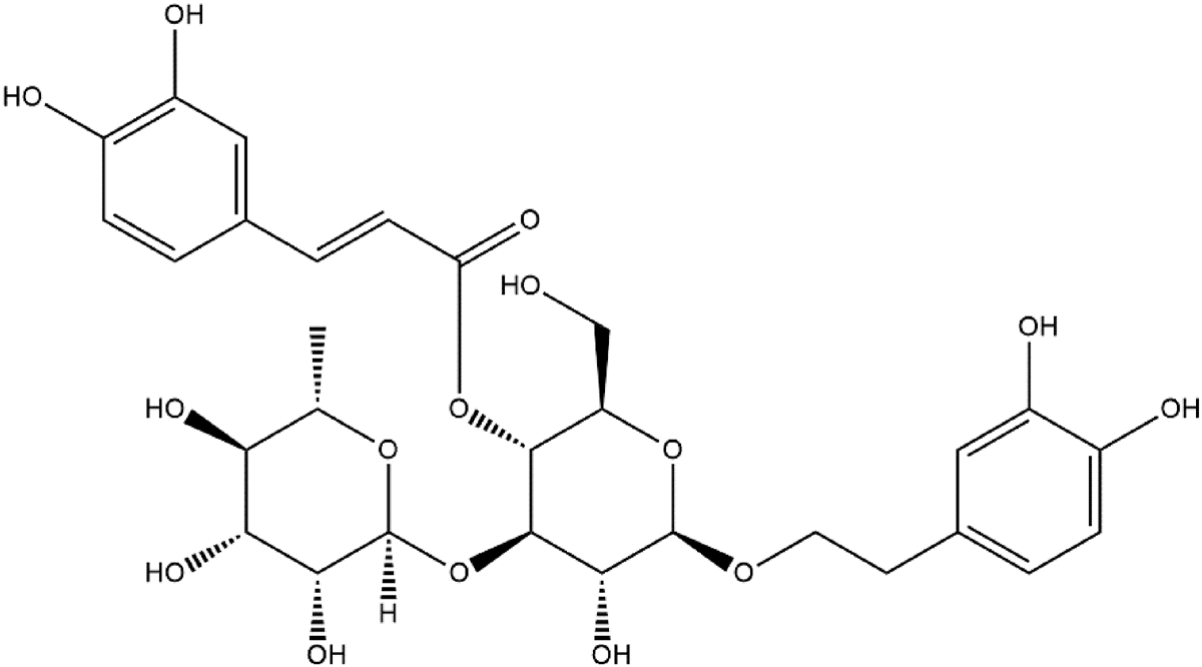
Structure of acteoside (Ac) from *C. cyrtophyllum* leaves.

*Clerodendrum cyrtophyllum* Turcz (Verbenaceae) is a deciduous shrub widely distributed in the south of China. In particular, Hainan Island has the most affluent wild population, known as Yangmieqing, Lubianqing, or Daqing. According to the Medicinal Botany record, the Chinese Folk Herbal Formula and the Practical Chinese Herbal Primary Color Chart, *C. cyrtophyllum* has been used as a folk medicine for the treatment of beriberi fever, jaundice, leukorrhea, syphilis, and typhoid^8^. Our research team has demonstrated that the ethanolic extracts and different solvent sub-fractions of *C. cyrtophyllum* leaves exhibit strong 2,2′-diphenyl-1-picrylhydrazyl (DPPH^.^) and 2,2′-azino-bis(3-ethylbenzothiazoline-6-sulphonicacid) (ABTS^.+^) scavenging activity, and the activity is positively correlated with the contents of phenolic compounds^9^. Further, acteoside (Ac), one of the twelve phenolic components identified from the EAF of *C. cyrtophyllum*, has been found to be a major component and show DPPH and ABTS radical scavenging activities, indicating it is principally responsible for the significant total antioxidant effect of *C. cyrtophyllum*^10^. Its structure contains several chemical groups, such as caffeic acid, 3,4-dihydroxyphenylethanol, glucose, and rhamnose (Fig. 1). Ac is also named verbascoside, and it was originally isolated from the flowers of *Syritnga vulgaris*. It has also been found in many other plants, such as *Paulownia tomentosa*, *Leucoseptrum japonicum*, *Forsythia viridissima*, *Rehmannia glutinosa*, and *Harpagophytum procumbens*^11^. Several studies have indicated that Ac exhibits various pharmacological activities, such as anti-tumor, anti-inflammatory, anti-nephritic, anti-oxidative, and hepatoprotective activities^12^. In addition, the antioxidant activity of Ac has been demonstrated to play an important role in its hepatoprotective effects^13^. Ac has been widely studied in oxidative stress models to evaluate physiological conditions in cells^14–16^.

In this study, a human hepatoma cell line (HepG2 cells) was used, because hepatocytes are the major site of xenobiotic metabolism within the liver and hence represent the most important cellular target for toxic reactive metabolites. Together, the ability of HepG2 cells to carry out biotransformation of xenobiotics, although with a lower metabolic capacity compared to hepatocytes, the absence of p53 mutations, and their easy usability in comparison to, for example, primary human hepatocytes, make these cells a convenient alternative for in vitro testing^17^. The ability of ethanolic crude extracts, various solvent fractions, and acteoside from *C. cyrtophyllum* leaves to protect HepG2 cells against *t*-BHP-induced oxidative damage has not been thoroughly investigated. we explored the in vitro cytoprotective and antioxidant activities of ethanolic crude extract, five different ethanol fractions (petroleum ether fraction (PEF), dichloromethane fraction (DMF), ethyl acetate fraction (EAF), *n*-butyl alcohol fraction (BAF) and the remaining fraction (RF)), and Ac from *C. cyrtophyllum* leaves in protecting HepG2 against *t*-BHP-induced oxidative stress.

## Results

### Effect of extracts on the viability of HepG2 cells under oxidative stress

**Figure 2:**
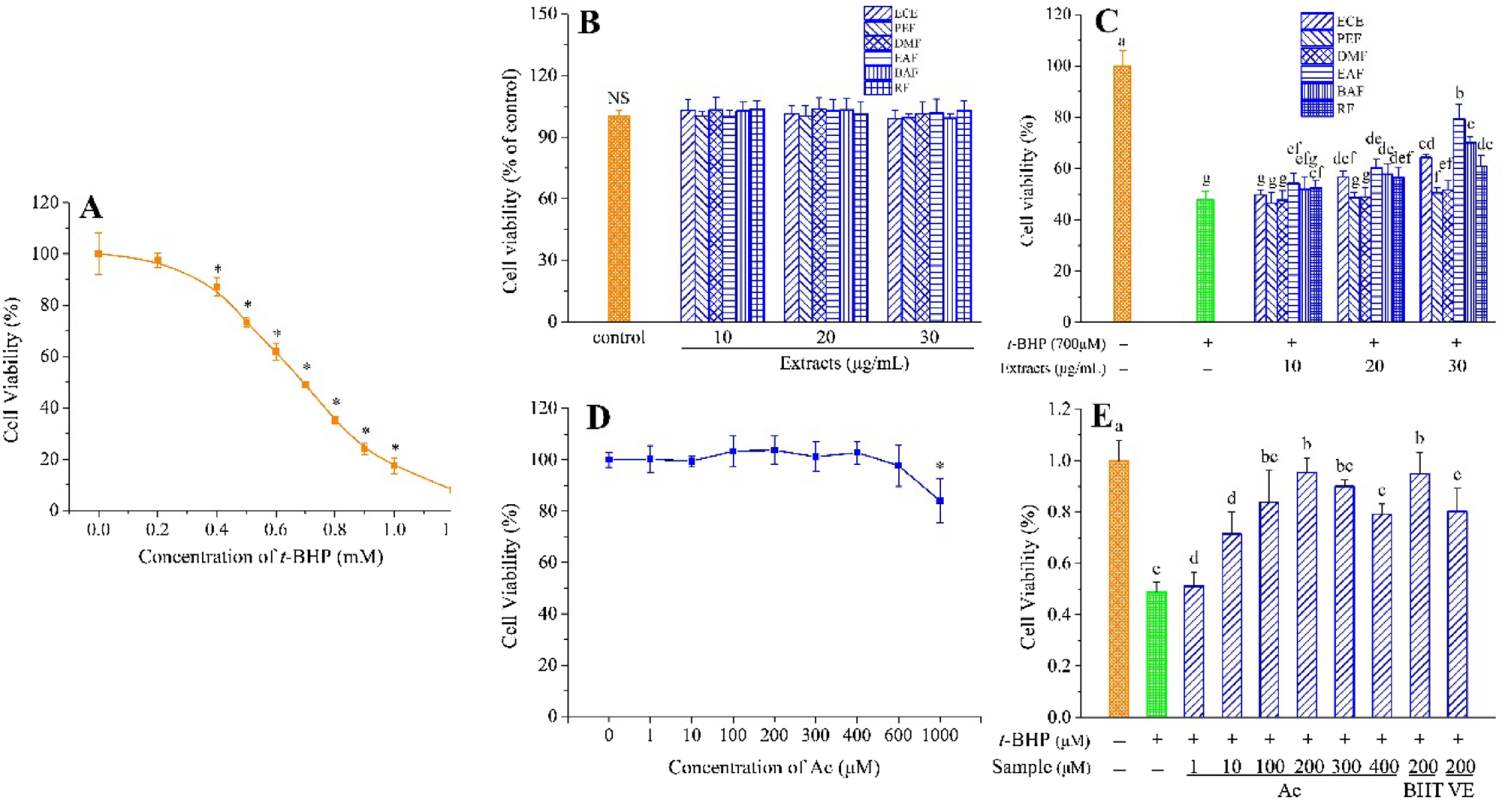
HepG2 cells viability. (A) Cells were treated with *t*-BHP at the indicated concentrations (0–1.2 mmol/L). *p < 0.05 compared with no *t*-BHP treatment. (**B**) Viability of HepG2 cells after treatment with different concentrations of ECE, PEF, DMF, EAF, BAF, RF. NS indicates that there is no significant difference between control and all sample groups (p < 0.05). (**C**) Cytoprotective effects of ECE, PEF, DMF, EAF, BAF, RF. (**D**) Viability of HepG2 cells after treatment with different concentrations of Ac. *p < 0.05 versus no Ac treatment. (**E**) Cytoprotective effects of Ac. Different letters above bars indicate significant difference (by ANOVA, p < 0.05). Result are expressed as mean ± SD (n ≥ 3). + , treated; −, untreated.

As shown in Fig. 2A, treatment with 700 μmol/L *t*-BHP for 3 h caused the viability of HepG2 cells to decrease to about 50.0%, compared with the control group; hence, this concentration was used to simulate oxidative damage in HepG2 cells in subsequent experiments.

Considering the extract itself may be toxic to cells, we determined the effects of all extracts (ECE, PEF, DMF, EAF, BAF, and RF) on the viability of HepG2 cells without the presence of *t*-BHP. The results show that the viability of extract-treated HepG2 cells was not significantly different from that of the control group (*p* > 0.05). The six extracts (at 10, 20, and 30 μg/mL) neither promoted nor decreased the viability of HepG2 cells (Fig. 2B). This finding indicates that all extracts are not cytotoxic and do not interfere with the cell viability assay of *t*-BHP.

We further evaluated the cytoprotective effects of the extracts against *t*-BHP-induced oxidative damage. As shown in Fig. 2C, treatment with 700 μmol/L *t*-BHP caused the survival rate of HepG2 cells to reduce by about 50%, compared to that of the control group. Additionally, the survival rate of HepG2 cells pretreated with ECE increased with increasing concentration of ECE. The survival rate of HepG2 cells treated with crude material at 20 and 30 μg/mL was significantly higher than that of *t*-BHP-treated group (*p* < 0.05). Furthermore, the survival rate of HepG2 cells pretreated with EAF, BAF, and RF increased with increasing sample concentration; however, both PEF and DMF at all concentrations had no significant effect on survival rate. At the same concentration of 30 μg/mL, the survival rate of HepG2 cells treated with EAF was significantly higher than that of the cells treated with other samples (*p* < 0.05), and the effect on cell viability can be ranked in the following order: RF < ECE < BAF < EAF.

### Effect of extracts on the accumulation of intracellular ROS

**Figure 3:**
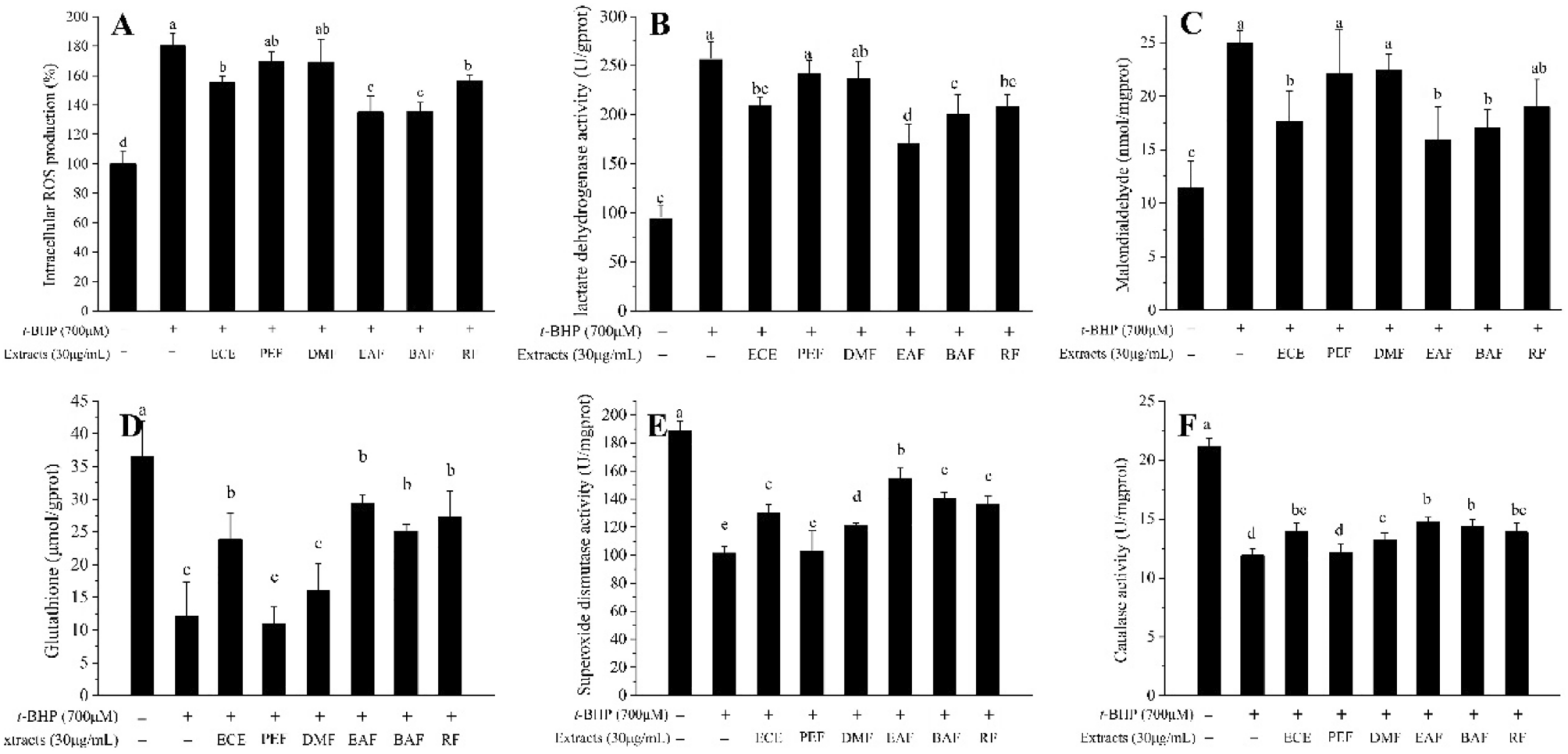
Effects of extracts on the indicators of HepG2 cellular antioxidant defense. (**A**) Effects of extracts on intracellular ROS. (**B**) Effects of extracts on extracellular lactate dehydrogenase activity. (**C**) Effects of extracts on extracellular malondialdehyde level. (**D**) Effects of extracts on GSH level. (**E**) Effects of extracts on SOD activity. (**F**) Effects of extracts on CAT activity. Different letters above bars indicate a significant difference (by ANOVA, p < 0.05). Results are expressed as mean ± SD (n ≥ 3). + , treated; −, untreated.

As illustrated in Fig. 3A, t-BHP clearly increased cellular ROS levels (by ~ twofold), but cells pretreated with ECE, EAF, BAF, and RF exhibited reduced ROS generation of 13.8%, 25.1%, 24.8% and 13.2% (*p* < 0.05), respectively, compared to *t*-BHP-stressed cells. In contrast, the reduction in ROS levels of PEF- and DMF-treated cells was not statistically significant. The above results demonstrate that ECE, EAF, BAF, and RF act as scavengers of the ROS generated by *t*-BHP in HepG2 cells.

### Effect of extracts on LDH release and MDA production

In order to evaluate the possible mechanism involved in the protective effect of *C. cyrtophyllum* extracts on cytotoxicity, LDH release, and MDA production were evaluated. Figure 3B,C shows that *t*-BHP-induced HepG2 cells pretreated with 100 μL of the 30 μg/mL *C. cyrtophyllum* extracts for 6 h exhibited LDH release and MDA level decreased. As compared with *t*-BHP-induced HepG2 cells (*p* < 0.05), indicating that ECE, EAF, BAF, and RF markedly attenuated oxidative stress of lipid peroxidation.

### Effect of extracts on GSH level and SOD and CAT activities

As shown in Fig. 3D, 700 μmol/L *t*-BHP significantly increased the cytoplasmic GSH level of the cells (*p* < 0.05); however, the GSH levels sharply decreased after the cells were treated with 100 μL of 30 μg/mL ECE, EAF, BAF, or RF for 6 h. In contrast, the GSH contents in the cells treated with PEF and DMF were not significantly different from those in the cells treated with *t*-BHP.

Furthermore, to characterize the antioxidant enzymes of HepG2 cells exposed to *t*-BHP, the activities of CAT and SOD were determined. As illustrated in Fig. 3E,F, pretreatment with ECE, PEF, DMF, EAF, BAF, or RF at 30 μg/mL significantly increased the activities of SOD and CAT (*p* < 0.05). EAF showed the highest SOD and CAT activities compared to that of cells treated with *t*-BHP. However, cells pretreated with PEF did not affect SOD and CAT activities.

### Effects of extracts on apoptosis protein caspase-3

**Figure 4:**
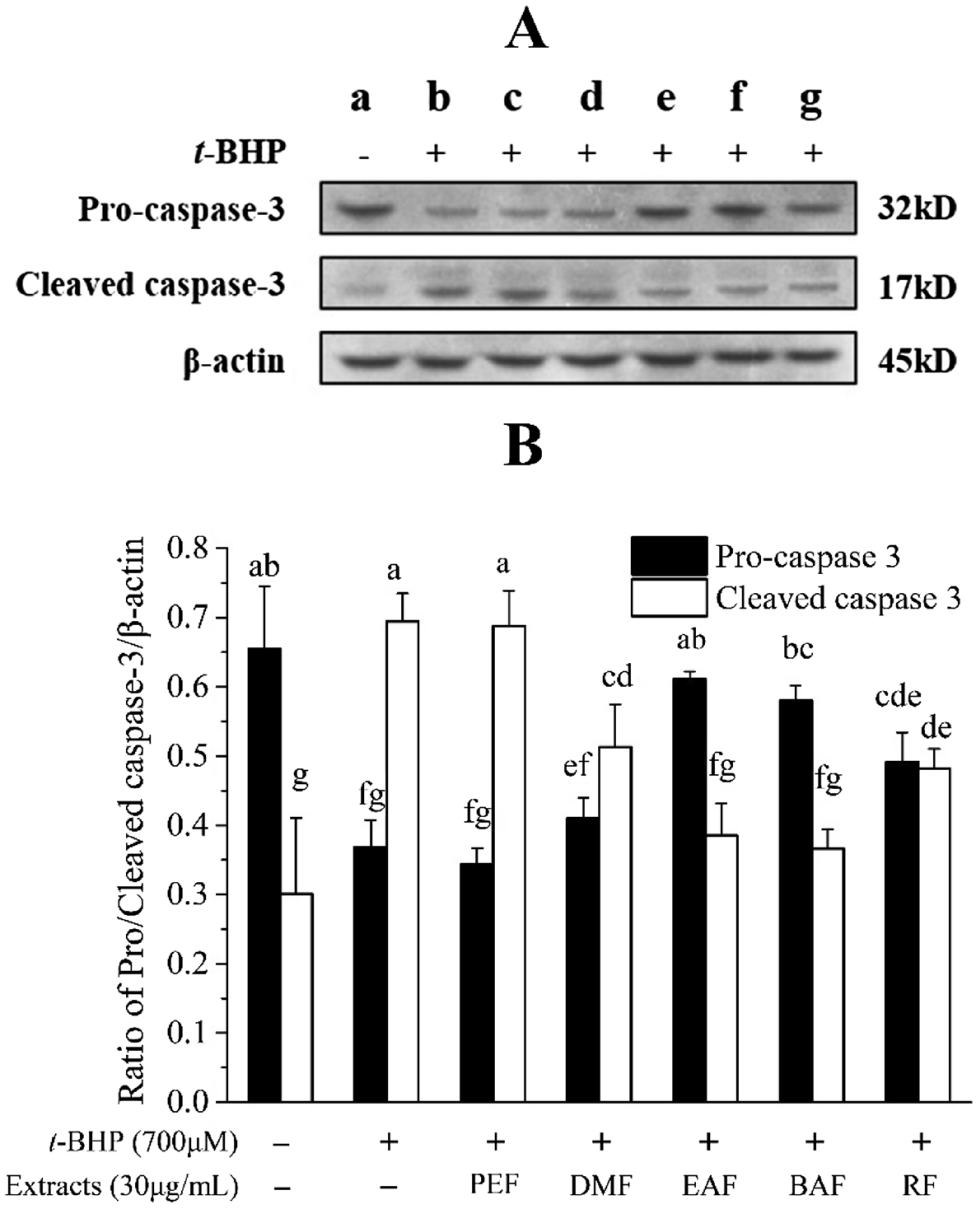
Effects of extracts on the expression of caspase-3 level in HepG2 cells. HepG2 cells were treated with 30 μg/mL samples for 6 h before being exposed to *t*-BHP (700 μmol/L) for 3 h. (**A**) The expressions of pro-caspase-3, cleaved caspase-3 and β-actin were used for normalization and verification of protein loading; (**B**) Quantitative pro-caspase-3 and cleaved caspase-3 expression after normalization to β-actin. (**a**) Control; (**b**) 700 μmol/L *t*-BHP treated; (**c**) 30 μg/mL PEF + 700 μmol/L *t*-BHP; (**d**) 30 μg/mL DMF + 700 μmol/L *t*-BHP; (**e**) 30 μg/mL EAF + 700 μmol/L *t*-BHP; (**f**) 30 μg/mL BAF + 700 μmol/L *t*-BHP; (**g**) 30 μg/mL RF + 700 μmol/L *t*-BHP. Different letters above bars indicate significant difference (by ANOVA, p < 0.05). Result are expressed as mean ± SD (n ≥ 3). + , treated; −, untreated.

Expression of caspase-3 in HepG2 cells was evaluated in our study, and the results are shown in Fig. 4A,B. Treatment with 700 μmol/L *t*-BHP significantly reduced the level of pro-caspase-3 (43.8%) and induced caspase-3 activation to 130.8%. As expected, pretreatment with EAF, BAF, and RF opposed these effects, and the pro-caspase-3 level significantly increased by 65.7%, 57.3%, and 33.4%, respectively. However, the level of cleaved caspase-3 significantly decreased by 44.5%, 50.1%, and 30.5%, respectively. It is important to note that pro-caspase-3 and cleaved caspase-3 levels of PEF- and DMF-treated cells were not significantly different from those in *t*-BHP-treated cells.

### Effect of Ac on the viability of HepG2 cells under oxidative stress

The cytotoxic effect of acteoside (Ac) on HepG2 cells is shown in Fig. 2D. After 6 h treatment with Ac at concentrations between 1 and 600 μmol/L, there was no significant toxicity in cell viability, compared to the control group. However, cell viability dropped slightly at 1000 μmol/L Ac. We further evaluated the cytoprotective effects of Ac against *t*-BHP-induced oxidative damage. As shown in Fig. 2E, pre-incubation of HepG2 cells with Ac (1–400 μmol/L) for 6 h reduced oxidative damage induced by *t*-BHP, increasing cell viability in a dose-dependent manner. This result was significantly different from that of the damage control. At the same concentration of 200 μmol/L, Ac and BHT promoted higher cell viability than VE against *t*-BHP-induced oxidative stress. These data demonstrate that Ac exhibits a remarkable cytoprotective capacity.

### Effect of Ac on intracellular ROS accumulation

**Figure 5:**
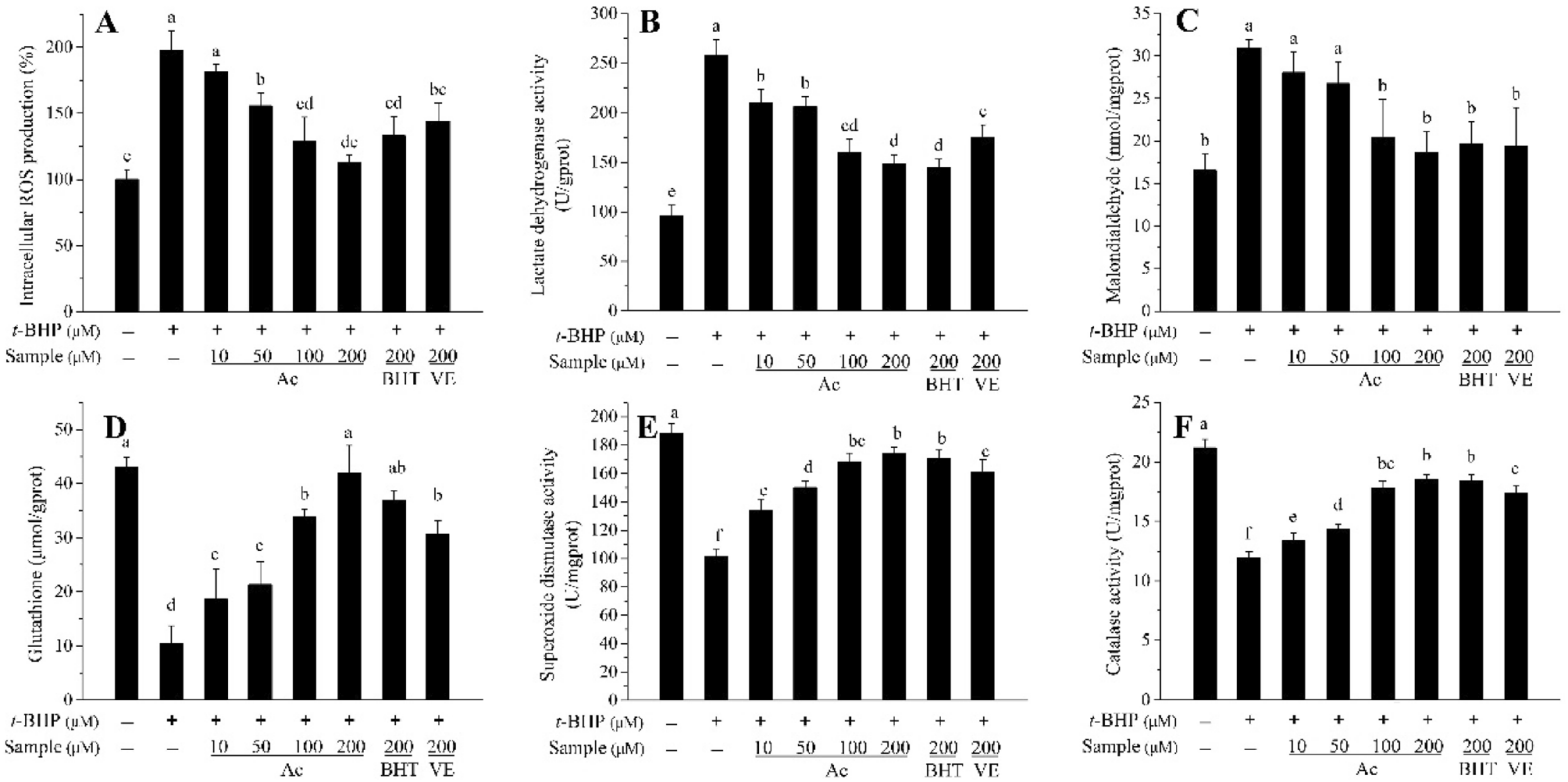
Effects of Ac, BHT, and VE on the indicators of HepG2 cellular antioxidant defense. (**A**) Effects of Ac, BHT, and VE on intracellular ROS. (**B**) Effects of Ac, BHT, and VE on extracellular lactate dehydrogenase activity. (**C**) Effects of Ac, BHT, and VE on extracellular malondialdehyde level. (**D**) Effects of Ac, BHT, and VE on GSH level. (**E**) Effects of Ac, BHT, and VE on SOD activity. (**F**) Effects of Ac, BHT, and VE on CAT activity. HepG2 cells were treated with 30 μg/mL samples for 6 h before being exposed to TBHP (700 μmol/L) for 3 h. ROS level, LDH activity, MDA level, GSH level, SOD activity, and CAT activity in HepG2 cells were detected through the kit. Different letters above bars indicate significant difference (by ANOVA, p < 0.05). Results are expressed as mean ± SD (n ≥ 3). Ac, acteoside; BHT, butylated hydroxytoluene; VE, vitamin E; + , treated; −, untreated.

The DCFH-DA fluorescence staining of ROS inhibition in *t*-BHP treated HepG2 cells is presented in Fig. 5A. Pretreatment with Ac (10–200 μmol/L) significantly reduced the fluorescence density, and the inhibition of ROS generation induced by *t*-BHP was proportional to the concentration of Ac, compared with the *t*-BHP treated group. The accumulation of ROS in the Ac-treated group was greater than in the positive controls BHT and VE at 200 μmol/L.

### Effect of Ac on LDH release and MDA level

As shown in Fig. 5B,C, pretreatment of HepG2 cells with Ac (10–200 μmol/L) for 6 h markedly decreased the release of LDH and production of MDA caused by *t*-BHP-induced HepG2 cell damage. The effect of the 100 μmol/L dose was similar to that of the standard BHT and VE.

### Effect of Ac on GSH level and SOD and CAT activities

To further determine the likely transcriptional mechanism underlying the protective effects of Ac, GSH level and the activities of intracellular antioxidant enzymes SOD and CAT were examined. As shown in Fig. 5D–F, exposure of HepG2 cells to 700 μmol/L *t*-BHP dramatically decreased the GSH level, as well as SOD and CAT activities. Pre-incubation of cells with 10–200 μmol/L Ac for 6 h attenuated these effects in a dose-dependent manner. At 200 μmol/L, Ac exhibited a greater increase in GSH and SOD and CAT activities than BHT and VE.

### Effects of Ac on apoptosis

**Figure 6:**
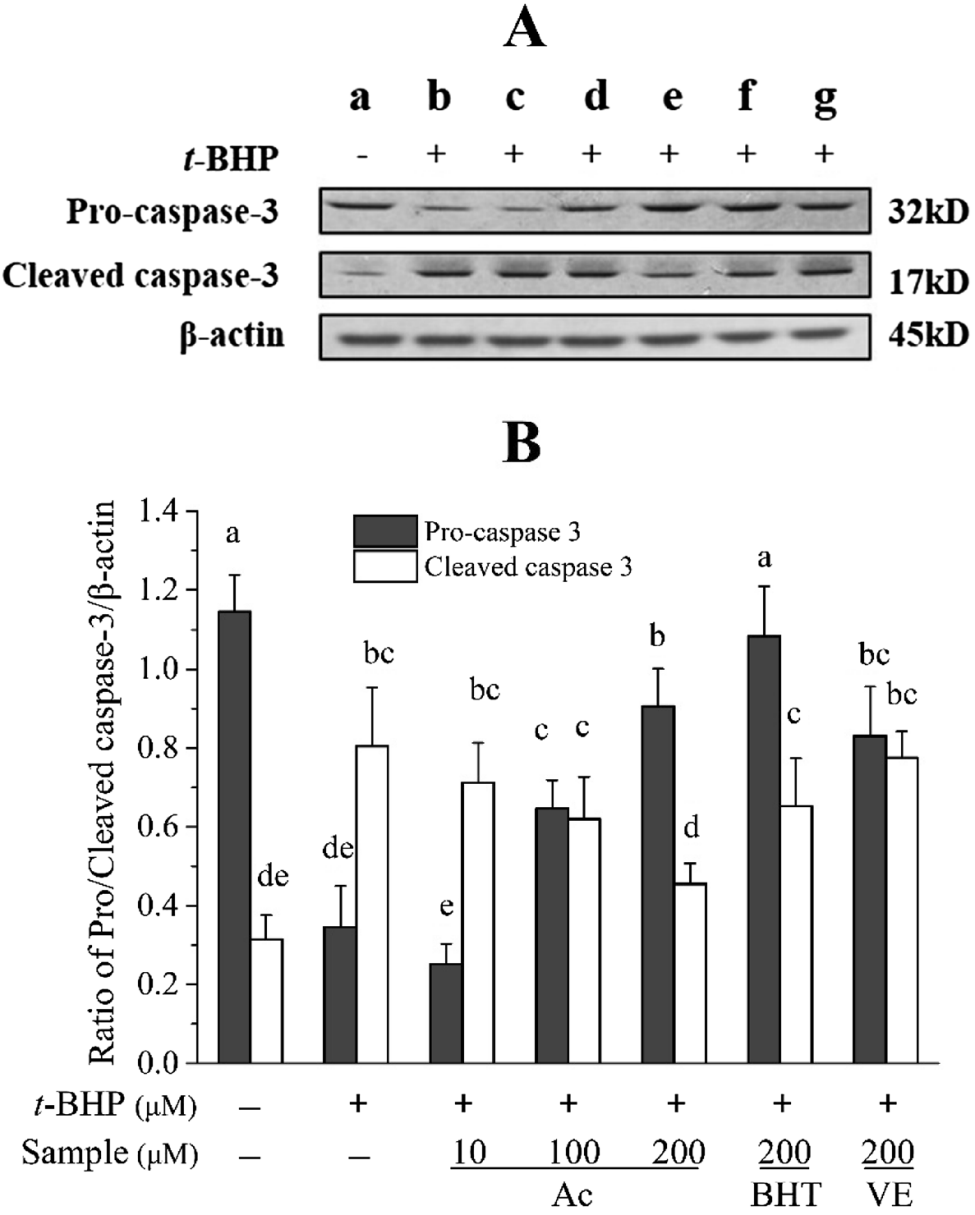
Effect of Ac, BHT, or VE on the expression of caspase-3 level in HepG2 cells. (**A**) The expressions of pro-caspase-3, cleaved caspase-3 and β-actin were used for normalization and verification of protein loading; (**B**) Quantitative pro-caspase-3 and cleaved caspase-3 expression after normalization to β-actin. (**a**) Control; (**b**) 700 μmol/L *t*-BHP treated; (**c**) 10 μmol/L Ac + 700 μmol/L *t*-BHP; (**d**) 100 μmol/L Ac + 700 μmol/L *t*-BHP; (**e**) 200 μmol/L Ac + 700 μmol/L *t*-BHP; (**f**) 200 μmol/L BHT + 700 μmol/L *t*-BHP; (**g**) 200 μmol/L VE + 700 μmol/L *t*-BHP. Different letters above bars indicate significant difference (by ANOVA, p < 0.05). Results are expressed as mean ± SD (n ≥ 3). + , treated; −, untreated.

To investigate the effect of Ac against the apoptosis response of HepG2 cells induced by oxidative stress, the expression of the apoptosis-related protein, caspase-3, was determined by western blot. As illustrated in Fig. 6A,B, treatment of HepG2 cells with Ac, followed by *t*-BHP, could increase the amount of anti-apoptotic pro-caspase-3 and reduce the amount of pro-apoptotic cleaved-caspase-3 in a dose-dependent manner.

**Figure 7:**
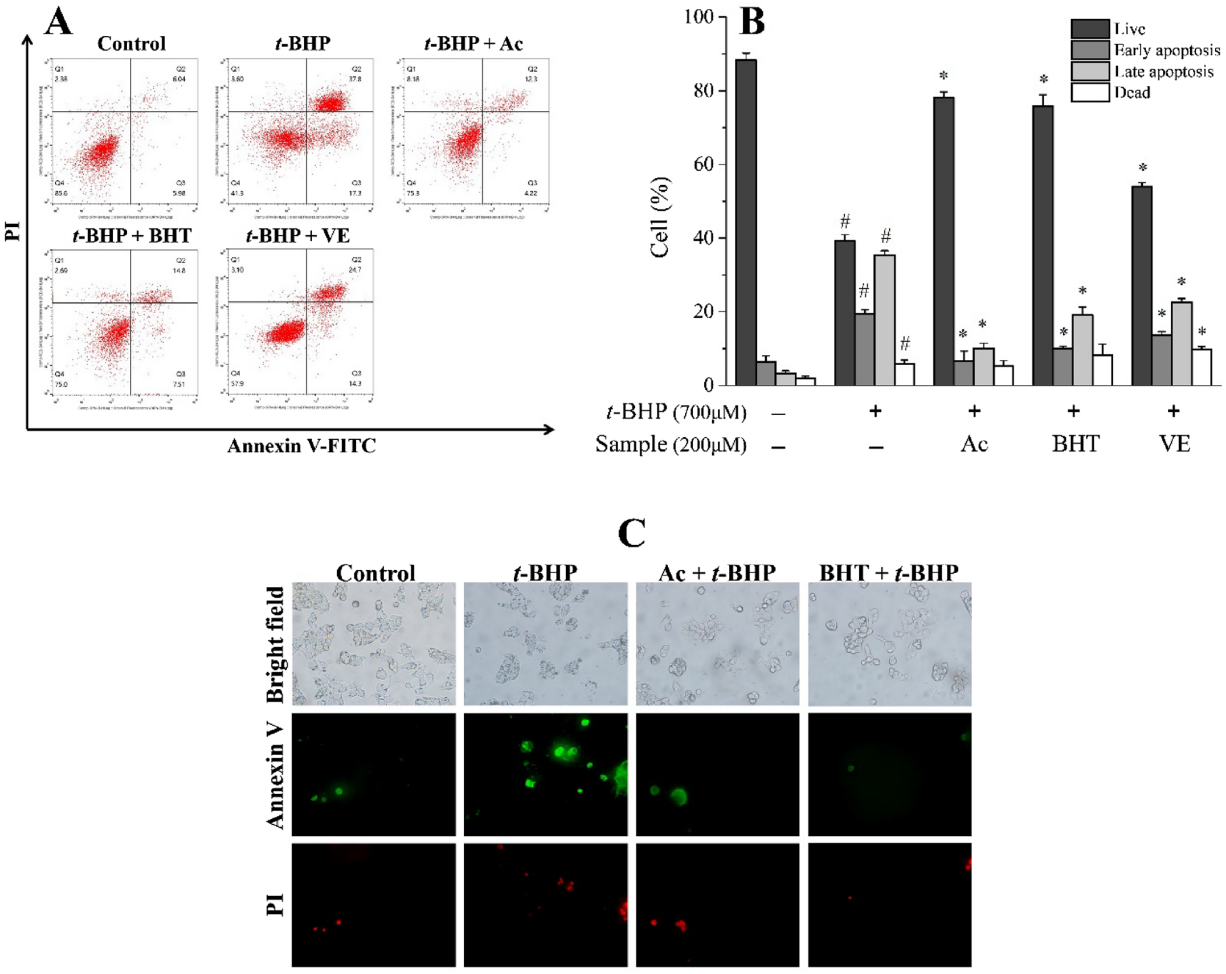
Effect of Ac, BHT and VE on apoptosis. (**A**) Representative dot pot of apoptosis evaluated by flow cytometric analysis after Annexin-V/PI double staining. Cells were gated based on size and granularity using FSC-A vs SSC-A to eliminate debris and clumped cells. The living cells and apoptotic cells were defined by the four-quarter gate setting tool in the software. (**B**) Quantitation results of apoptosis evaluated by flow cytometric analysis. (**C**) Fluorescence micrographs to assess Annexin-V/PI double staining of HepG2 cells. Results are expressed as mean ± SD (n ≥ 3). #p < 0.05 versus the same state of control, *p < 0.05 versus TBHP treatment. + , treated; −, untreated.

Apoptotic cell death was further quantified by Annexin V-FITC/PI flow cytometry. As illustrated in Fig. 7A–C, HepG2 cells treated with Ac demonstrated Annexin-V positive cells were inhibited in a time-dependent mannerand a distinct population decline in the upper right quadrant, directly indicating Ac can prevent *t*-BHP-triggered apoptosis in HepG2 cells.

## Discussion

Oxidative stress is an essential factor that causes many chronic diseases, and it is closely related to the accumulation of ROS. For example, the liver disease usually results in excess ROS production in the body^18^. Proper use of antioxidants is of great significance in preventing oxidative-related disorders and, in some cases, helps in their treatment^19^. Phytochemical or antioxidant therapies are considered promising strategies to protect cells from oxidative damage, and plant-derived natural products are important sources of antioxidants^20^. Various studies have screened different plant extracts for their antioxidant potential^15,21,22^. Particularly, polyphenols have proven their efficacy as antioxidants and have been adopted in the prevention and/or treatment of various human diseases^23^. In this study, we demonstrated the antioxidant potential and cytoprotective effects of ethanolic crude extract from *C. cyrtophyllum* leaves (ECE) and five derived fractions, PEF, EAF, BAF, and RF, as well as polyphenolic Ac (Fig. 1). The results furthermore confirmed the antioxidant effects of *C. cyrtophyllum* extracts^9^.

High levels of dietary compounds can be toxic and mutagenic in cell culture systems^24^. None of the extracts (ECE, PEF, DMF, EAF, BAF, and RF) at 30 μg/mL were significantly toxic to HepG2 cells viability compared to the control group (Fig. 2A). In accordance with a previous report on the physiological effects of Ac^25^, our study found Ac was not toxic in cellular physiological contexts. However, Ac did increase the oxidative load, affect the activity of proteostatic modules, and suppress matrix metalloproteinases in tumor cell lines. *t*-BHP is often used to stimulate oxidative stress responses in HepG2 cells^17,26^. In the present study, we observed that the treatment of cells with *t*-BHP caused an increase of intracellular ROS content and resulted in decreased viability of HepG2 cells (Fig. 2B), but incubation with ECE or different polar extracts could mitigate apoptosis (Fig. 2C).

Oxidative damage in hepatocytes was induced by the treatment of *t*-BHP, an organic hydroperoxide that can be metabolized to free radicals, making it a pivotal contributor to intracellular oxidative stress^22^. The antioxidant capacity can be evaluated by measuring the ability to scavenge ROS in *t*-BHP oxidative damage cell models^3^. Breakdown of the endogenous antioxidant defense mechanism can be reflected by the amount of LDH released, MDA levels, GSH levels, and SOD and CAT activities^27,28^. According to our results, after HepG2 cells were exposed to ECE, the amount of LDH released and the MDA level decreased. Conversely, GSH levels, SOD activity, and CAT activity significantly increased, indicating that ECE can effectively remove intracellular ROS. Similar results were also observed in EAF, BAF, and RF; and EAF was the most effective among all extracts (Fig. 3A–F). It is possible that EAF contains high concentrations of phenols (the major antioxidant constituents of *C. cyrtophyllum*), which have antioxidant activity and can be dissolved in a polar solvent^8^.

Oxidative stress plays a role as a common mediator to provoke cytosolic reaction cascades involving caspases and regulatory factors, such as caspase-3. Caspase-3 is a key terminal cleavage enzyme in the process of apoptosis, eventually leading to caspase-dependent and caspase-independent cell death^29,30^. In the present study, HepG2 cells were treated with 30 μg/mL *C. cyrtophyllum* extracts for 6 h before being exposed to *t*-BHP (700 μmol/L) for 3 h. The expression of caspase-3 was using measured western blot. ECE, especially for EAF, BAF, and RF, significantly up-regulated the expression of pro-caspase-3 and down-regulated the expression of cleaved caspase-3 (Fig. 4A,B). It is believed that ECE may interact with pro-caspase-3 to prevent cleavage by caspase-3.

To further investigate the underlying endogenous antioxidant defense mechanisms of *C. cyrtophyllum*, Ac was found to be a significant component (0.803 g, 0.54%) isolated from EAF. Reportedly, Ac exerts protective effects against 1-methyl-4-phenylpyridinium ion (MPP^+^)-induced oxidative stress in pheochromocytoma (PC12) and SH-SY5Y neuronal cells^31,32^. It is capable of reducing the levels of aspartate aminotransferase (AST), alanine aminotransferase (ALT), and MDA in the liver of mice injured by Bacillus Calmette-Guerin (BCG)/LPS^33^. Wang and coworkers^34^ demonstrated the protective effects of Ac on amyloid β-protein-induced human neuroblastoma SH-SY5Y cell injury by inhibiting caspase-3 activation. In this study, Ac showed no apparent toxic or promotional effects in physiological cellular contexts at 1–600 μmol/L and significantly increased the viability of *t*-BHP-induced HepG2 cells (Fig. 2D,E). We found that 700 μmol/L *t*-BHP elevated intracellular ROS, leading to a roughly two-fold increase in fluorescence intensity. Pretreatment with Ac (10–200 μmol/L) for 6 h greatly reduced the production of ROS (Fig. 5A). Additionally, it reduced the amount of LDH released (Fig. 5B), as well as MDA levels (Fig. 5C), but it increased GSH levels (Fig. 5D), SOD activity (Fig. 5E), and CAT activity (Fig. 5F), compared to the positive control BHT and VE. Thus, it is evident that Ac can reduce oxidative stress damage in cells by inhibiting the production of ROS, reducing the production of peroxidation products, and enhancing the activity of antioxidant enzymes. Furthermore, Ac could up-regulate pro-caspase-3 while down-regulating cleaved caspase-3, which is evidenced by the ability of Ac to suppress TBHP-induced HepG2 cells apoptosis (Fig. 6A,B). Flow cytometry data and fluorescence micrographs provide reliable and clearly distinguished results between subpopulation of apoptotic cells and were closely intercorrelated^35^. We found that Ac enhanced the viability of HepG2 cells under oxidative stress and reduced the number of early and late apoptotic cells. (Fig. 7A–C). These results are in good agreement with the effects of Ac in preventing apoptosis.

## Conclusions

In conclusion, we demonstrated that ethanol crude extracts from *C. cyrtophyllum* leaves could reduce the oxidative damage caused by *t*-BHP to HepG2 cells. Among all derived fractions, ethyl acetate fraction had the highest protective effect, followed by *n*-butanol fraction and the water fraction. The extracts could slow down the apoptosis of mitochondria by inhibiting the production of ROS. Moreover, enhanced levels of antioxidants and elevated activities of antioxidant enzymes in cells were also measured. Ac is naturally found in *C. cyrtophyllum* leaves, and it inhibits the production of ROS and prevents apoptosis by inhibiting the activation of caspase-3. Ac has shown an excellent cytoprotective effects and antioxidant activities against *t*-BHP induced oxidative damage, equal to or better than synthetic antioxidants BHT and natural antioxidants VE. Ac may be the main active component of *C. cyrtophyllum*, with desirable antioxidant activity and the potential to develop into plant originated antioxidant.

## Materials and methods

### Plant material

Leaves of *C. cyrtophyllum* (No. 201702-DQ) were collected at a very limited scale (1 kg) surrounding the Dead Crater Garden on Hainan Island, China. They were identifed by Prof. Xiaobo Yang of College of Ecology and Environment, Hainan University. The People’s Republic of China issued the specific permissions are required from authority of plant collection in a protected area of land, but not a national geological garden. The location we collect our plant materials is a national geological garden and the author was not obliged to have any permissions. This work did not involve endangered or protected species, the species *C. cyrtophyllum* is a common plant growing nearby the curbside. The study’s authors promise the use of plants in the present study complies with international, national and/or institutional guidelines. A voucher specimen of the plant (P-DQ001) was deposited in the herbarium of Laboratory of Tropical Pharmaceutical Biomolecules, School of Chemical Engineering and Technology, Hainan University.

### Preparation of extracts and acteoside

The ethanol extract of *C. cyrtophyllum* leaves (ECE) was first extracted with 49% ethanol for 85 min, using ultrasonic-assisted extraction. ECE was then subjected to the following extractions: ECE was extracted with pure petroleum ether to obtain petroleum ether fraction (PEF); ECE was extracted with pure dichloromethane to obtain dichloromethane fraction (DMF); ECE was extracted with pure ethyl acetate to obtain ethyl acetate fraction (EAF); ECE was extracted with n-butanol to obtain n-butanol fraction (BAF). The remaining ECE was a residual aqueous fraction (RF). All extracts were frozen, dehydrated, and stored at − 20 °C. Acteoside was isolated from the EAF fraction using column chromatography. All extracts, Ac, butylated hydroxytoluene (BHT) vitamin E (VE) were dissolved in dimethyl sulfoxide (DMSO) and diluted with FBS-free medium to the required concentrations. The mixture was applied to HepG2 cells. The final DMSO concentration was kept below 0.1% (v/v).

### Cell Culture and treatment

HepG2 cells were purchased from the Type Culture Collection of the Chinese Academy of Sciences (Shanghai, China). The cells were cultured in MEM medium containing 10% fetal bovine serum, penicillin (100 units/ml), and streptomycin (100 μg/ml) at 37 ℃ in an incubator humidified with 5% CO_2_. For in vitro experiments, HepG2 cells were exposed to various concentrations of *t*-BHP (0, 200, 400, 500, 600, 700, 800, 900, 1000, 1200 μmol/L) for 3 h to induce oxidative stress, or were pretreatment with indicated concentrations of extracts, acteoside, BHT or VE for 6 h prior to treatment with 700 μmol/L *t*-BHP for a further 3 h.

### Cell viability assay

Cell viability was determined by MTT assay. HepG2 cells were seeded in a 96-well plate at a density of 1 × 10^5^ cells/well and incubated at 37 °C for 24 h. The cells were then treated with the above extracts for 6 h. Subsequently, 0.5 mg/mL MTT reagent dissolved in MEM was added to each well, and the plate was further incubated at 37 °C for 3 h. After the culture medium was discarded, the cells were washed once with PBS (pH 7.2), and DMSO was added to solubilize the formazan product. Absorbance was then measured at 570 nm using a micro-plate reader (KeHua ST-360, Shanghai, China).

### Measurement of intracellular ROS

The effects of the extracts, acteoside, BHT, and VE on the production of *t*-BHP-induced ROS in HepG2 cells were examined using DCFH-DA fluorescence. HepG2 cells were incubated with various doses of the extracts or compounds for 6 h. They were treated with 700 μmol/L *t*-BHP for 3 h, followed by DCFH-DA (10 μmol/L) for an additional 30 min. The cells were then washed three times with bacteria-free PBS. Fluorescence intensity of the cells was determined using a multi-mode microplate reader (Tecan, Männedorf, Switzerland) at an excitation wavelength of 488 nm and an emission wavelength of 525 nm.

### Determination of LDH, MDA, GSH, and antioxidant enzyme activities

The release of LDH into the culture medium was measured using an LDH assay kit (A020-2, Jiancheng Bioengineering Institute, Nanjing, China). Levels of MDA and GSH, as well as the activities of SOD and CAT, in the cells were measured using the corresponding assay kits (A003-4, A006-2, A001-3, A007-1, Jiancheng Bioengineering Institute, Nanjing, China) according to the manufacturer’s instructions.

### Western blot analysis

Western blot analysis was performed following the method described by Liu et al.^23^ with slight modification. HepG2 cells were cultured in 6-well plates and treated with samples and *t*-BHP, as described above. After treatment, the cells were washed three times with PBS and were treated with RIPA lysate in a refrigerator (4 °C) for 2 h. The samples were then centrifuged at 12,000 rpm for 10 min. The lysate was collected, and the concentration of protein in the supernatant was determined using BCA assay. Proteins were first separated by sodium dodecyl sulfate–polyacrylamide gel electrophoresis (SDS-PAGE) using 12% polyacrylamide gel. The protein bands were transferred to a polyvinylidene fluoride (PVDF) membrane. After incubating with 5% skim milk in TBST buffer for 2 h at room temperature on a shaker, the membrane was incubated with primary antibody for 2 h at room temperature. Subsequently, the membrane was washed three times with TBST buffer for 5 min each and was then incubated with a secondary antibody for 1 h at room temperature. After the membrane was washed three times with TBST buffer, the bands on the membrane were visualized using an ECL system; the band density was optically scanned using ImageJ software.

### Annexin V-FITC/PI double staining

HpeG2 cells were seeded in 6-well plates and incubated with or without acteoside, BHT, and VE at various doses for 6 h. The cells were then treated with 700 μmol/L *t*-BHP for 3 h. After washing with PBS, the cells were dissolved in 500 μL of 1 × binding buffer and stained with 5 μL Annexin-V and 10 μL PI (Multisciences Biotech, Hangzhou, China). Fluorescence of the cells was measured at an excitation wavelength of 488 nm and an emission of 530 nm using a flow cytometer (Merck kGaA, Darmstadt, Germany). The voltage of FSC, SSC and fluorescence channels were adjusted by blank tube, and under this voltage condition, the compensation of fluorescence channel was adjusted by single dye tube. After acquisition, data were exported and analyzed using FlowJo version 10. The cells were also observed under a BX51-TRF fluorescence microscope (Olympus). FITC and Rhodamine channels on this fluorescence microscope were used for observation.

## Supplementary Information

Supplementary Information.

## Data Availability

All data generated or analysed during this study are included in this published article and its supplementary information files.
