# Supplementary material for: Cytoprotective effects and antioxidant activities of acteoside and various extracts of Clerodendrum cyrtophyllum Turcz leaves against t-BHP induced oxidative damage
Source: Sci Rep. 2022 Jul 25;12:12630. doi: 10.1038/s41598-022-17038-w (PMC9314432; doi:10.1038/s41598-022-17038-w)
Supplement: Supplementary file 1 — Supplementary Information. [file 41598_2022_17038_MOESM1_ESM.docx]

**Supplementary Information**

**Cytoprotective effects and antioxidant activities of acteoside and various extracts of Clerodendrum cyrtophyllum Turcz leaves against t-BHP induced oxidative damage**

Junjie Zhu^a^, Gang Li^a^, Jing Zhou^b^, Zhiyong Xu^b^, Jing Xu^a,^*

*^a^ School of Chemical Engineering and Technology, Hainan University, Haikou 570228, People’s Republic of China*

*^b^ School of Life and Pharmaceutical Sciences, Hainan University, Haikou 570228, People’s Republic of China*

^*^ To whom correspondence should be addressed.

Prof. Dr. Jing Xu, Tel.: ++86–898–6627–9226, Fax: ++86–898–6627–9010, E–mail: [happyjing3@163.com](mailto:happyjing3@163.com)

.

**Experimental Section**

[Figure S1. ^1^H-NMR of acteoside. 1](#_Toc106407702)

[Figure S2. ^13^C-NMR of acteoside. 2](#_Toc106407703)

[Figure S3. ^1^H-^1^H COSY of acteoside. 3](#_Toc106407704)

[Figure S4. HMQC of acteoside. 4](#_Toc106407705)

[Figure S5. HMBC of acteoside. 5](#_Toc106407706)

[Figure S6. (+) ESI-MS of acteoside. 6](#_Toc106407707)

[Figure S7. (-) ESI-MS of acteoside. 7](#_Toc106407708)

[Figure S8. Effect of ECE, PEF, DMF, EAF, BAF and RF on the expression of caspase-3 levels in HepG2 cells. 9](#_Toc106407709)

[Figure S9. Effect of Ac on the expression of caspase-3 levels in HepG2 cells. 11](#_Toc106407710)

[Table S1. Effect of t-BHP on HepG2 cell viability. 12](#_Toc106407711)

[Table S2. Cytotoxicity of ECE, PEF, DMF, EAF, BAF and RF on HepG2 cells. 12](#_Toc106407712)

[Table S3. The protective effect of ECE, PEF, DMF, EAF, BAF and RF on t-BHP -induced oxidative damage. 13](#_Toc106407713)

[Table S4. Cytotoxicity of Ac on HepG2 cells. 13](#_Toc106407714)

[Table S5. The protective effect of Ac on t-BHP -induced oxidative damage. 14](#_Toc106407715)

[Table S6. Effect of ECE, PEF, DMF, EAF, BAF and RF on HepG2 intracellular ROS. 14](#_Toc106407716)

[Table S7. Effects of ECE, PEF, DMF, EAF, BAF and RF on LDH level. 15](#_Toc106407717)

[Table S8. Effects of ECE, PEF, DMF, EAF, BAF and RF on MDA level. 15](#_Toc106407718)

[Table S9. Effects of ECE, PEF, DMF, EAF, BAF and RF on GSH level. 15](#_Toc106407719)

[Table S10. Effects of ECE, PEF, DMF, EAF, BAF and RF on SOD activity. 16](#_Toc106407720)

[Table S11. Effects of ECE, PEF, DMF, EAF, BAF and RF on CAT activity. 16](#_Toc106407721)

[Table S12. Effect of Ac on HepG2 intracellular ROS. 16](#_Toc106407722)

[Table S13. Effects of Ac on LDH level. 17](#_Toc106407723)

[Table S14. Effects of Ac on MDA level. 17](#_Toc106407724)

[Table S15. Effects of Ac on GSH level. 17](#_Toc106407725)

[Table S16. Effects of Ac on SOD activity. 18](#_Toc106407726)

[Table S16. Effects of Ac on CAT activity. 18](#_Toc106407727)

[Table S17. Data of Figure 4 analyzed by ImageJ software. 18](#_Toc106407728)

[Table S18. Data of Figure 6 analyzed by ImageJ software. 19](#_Toc106407729)


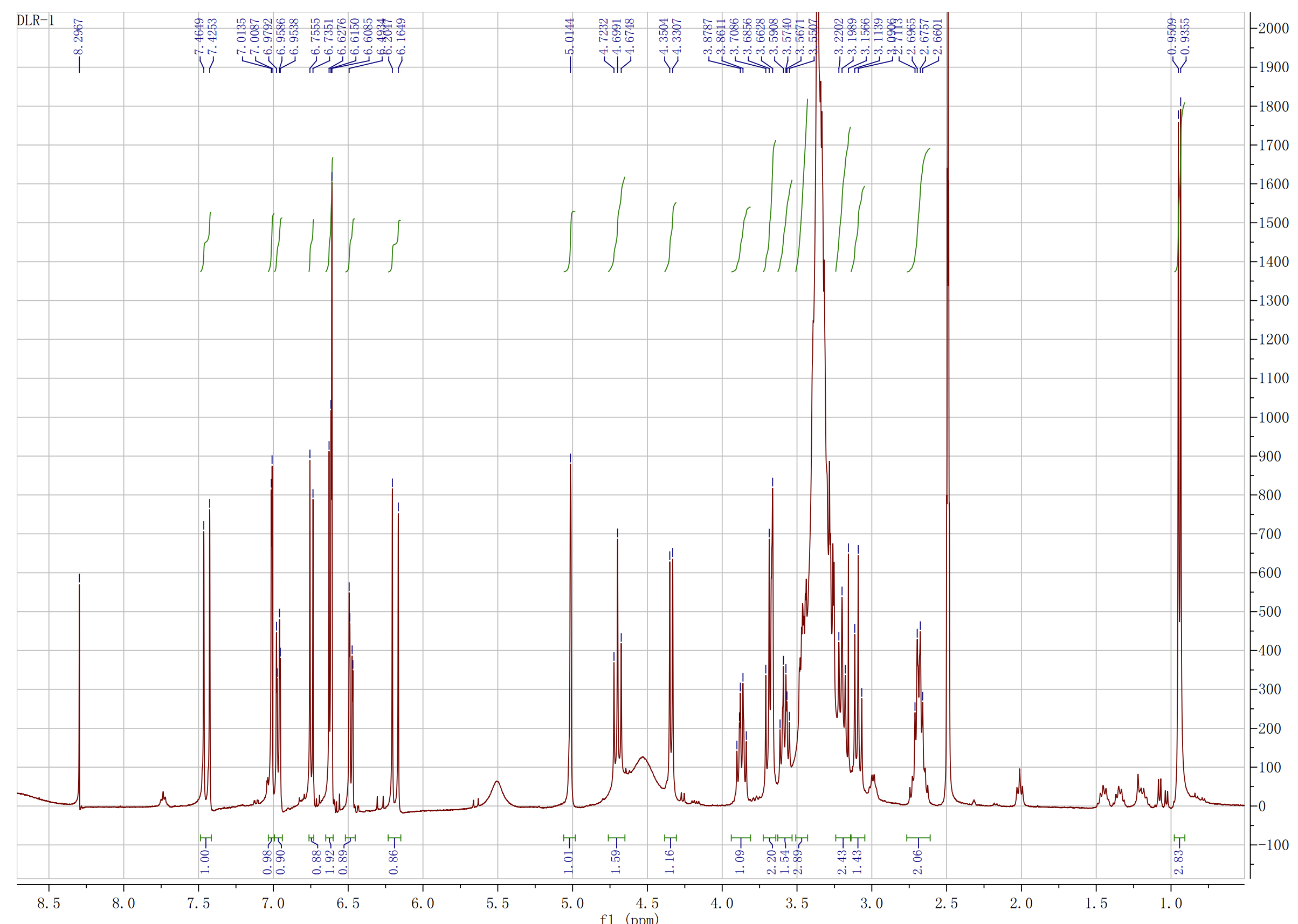


# Figure S1. ^1^H-NMR of acteoside.


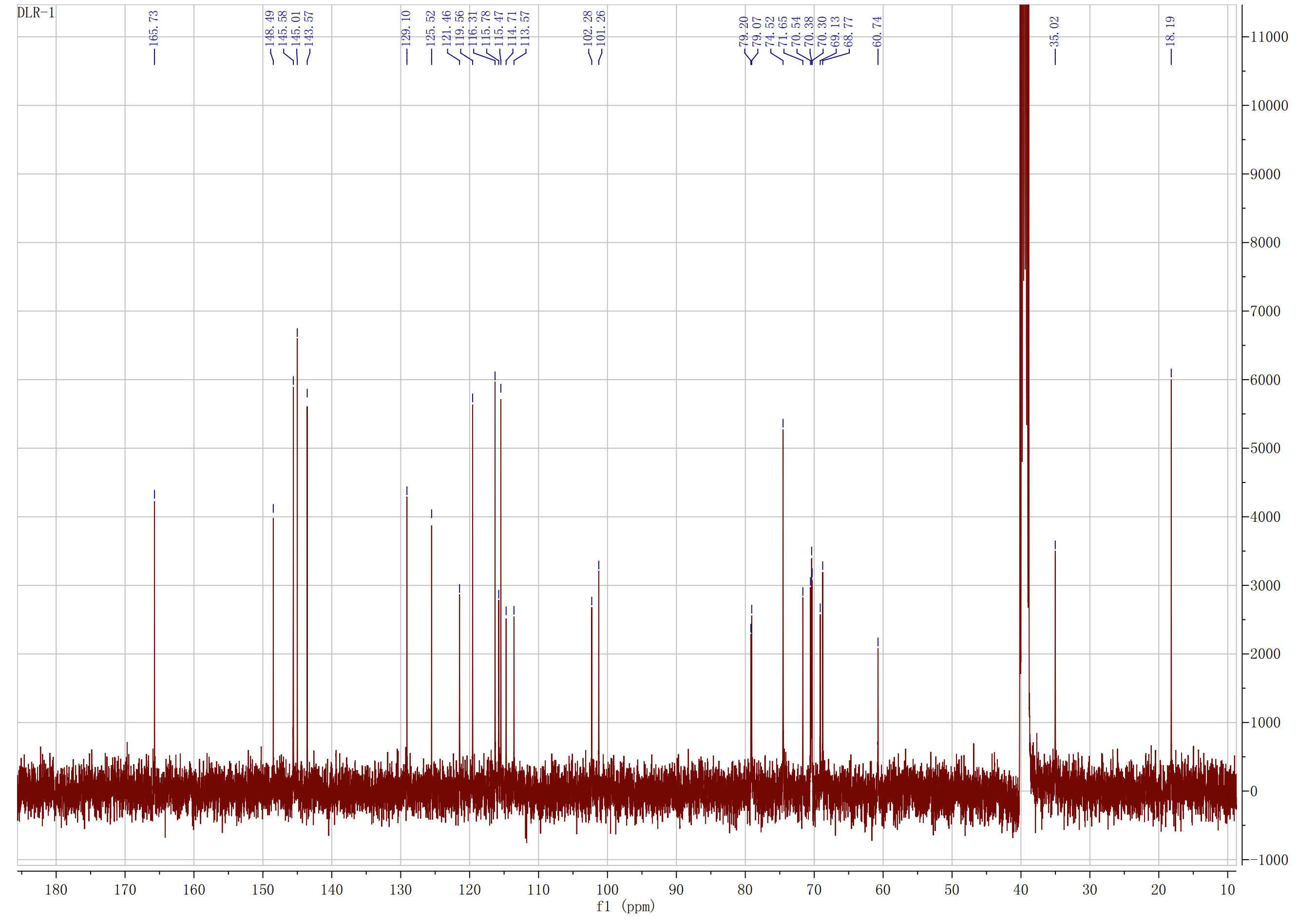


# Figure S2. ^13^C-NMR of acteoside.


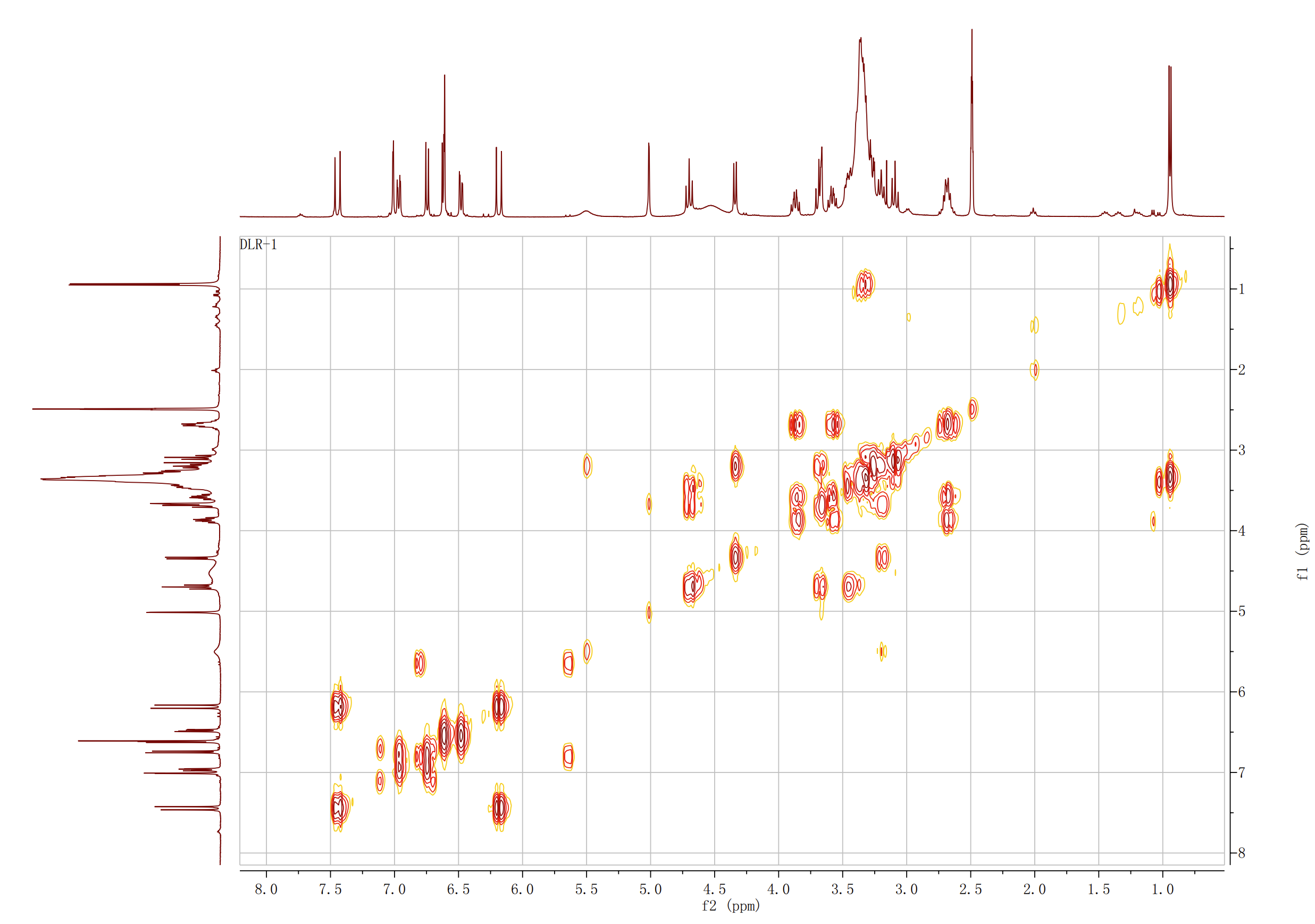


# Figure S3. ^1^H-^1^H COSY of acteoside.


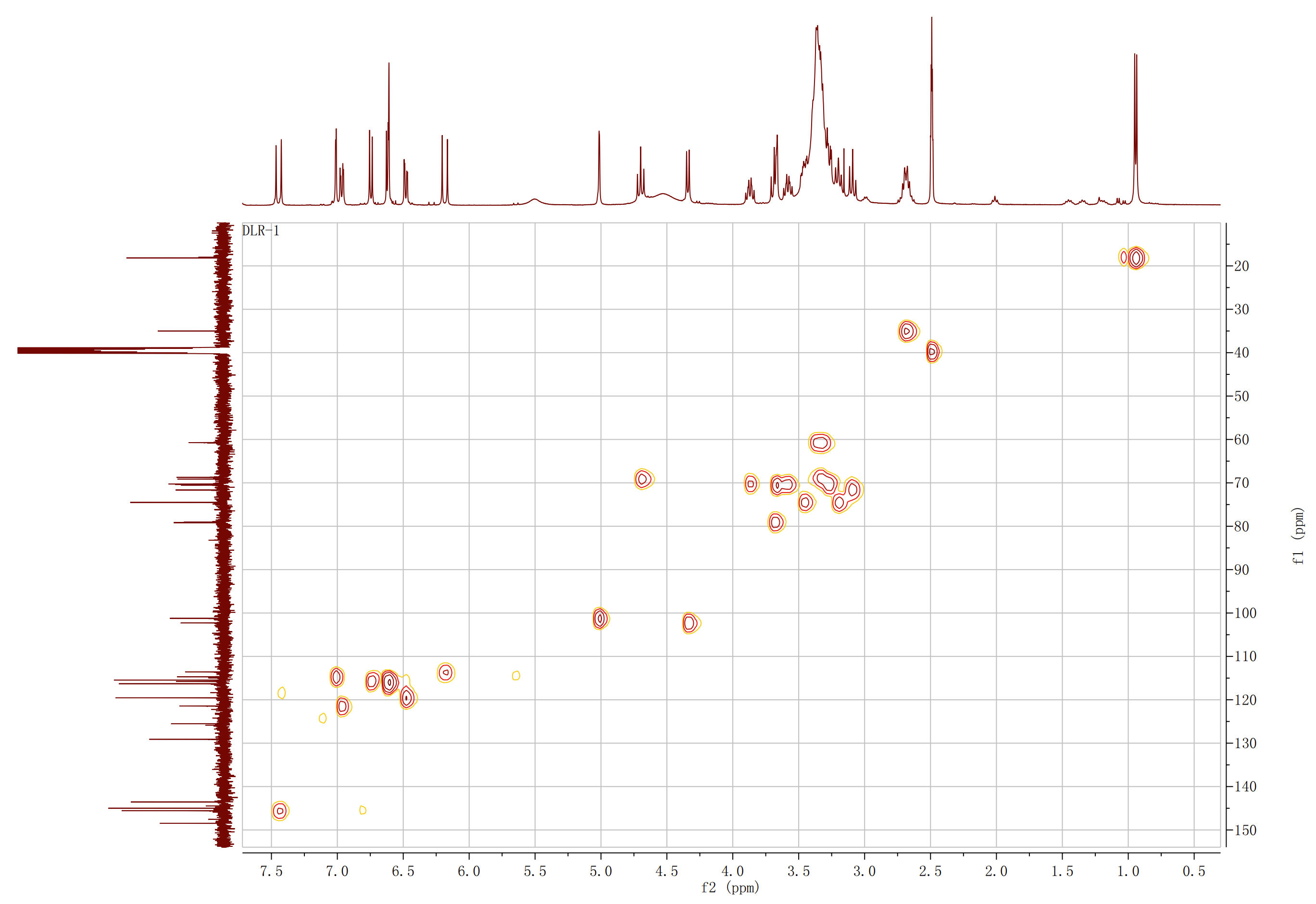


# Figure S4. HMQC of acteoside.


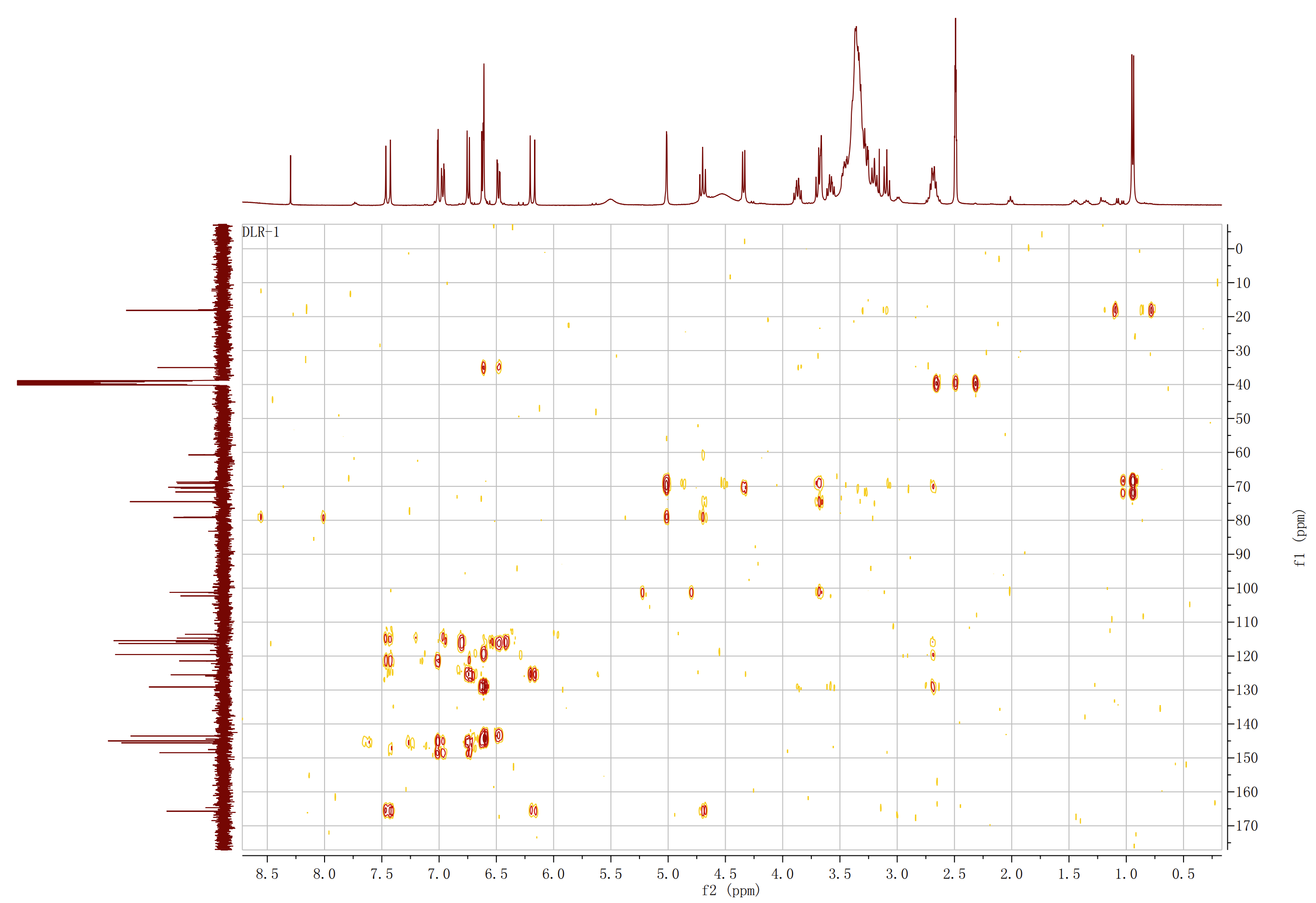


# Figure S5. HMBC of acteoside.


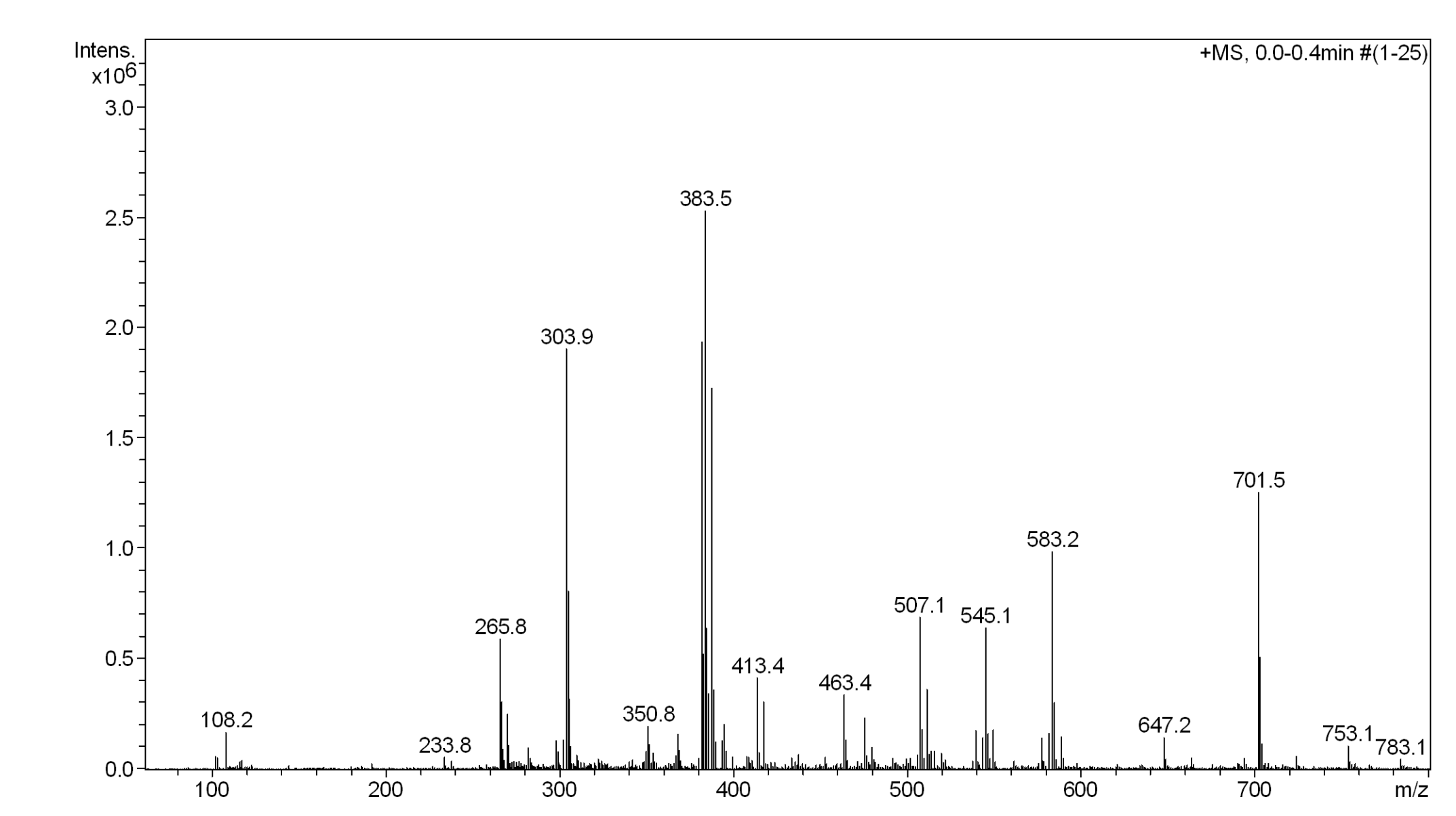


# Figure S6. (+) ESI-MS of acteoside.


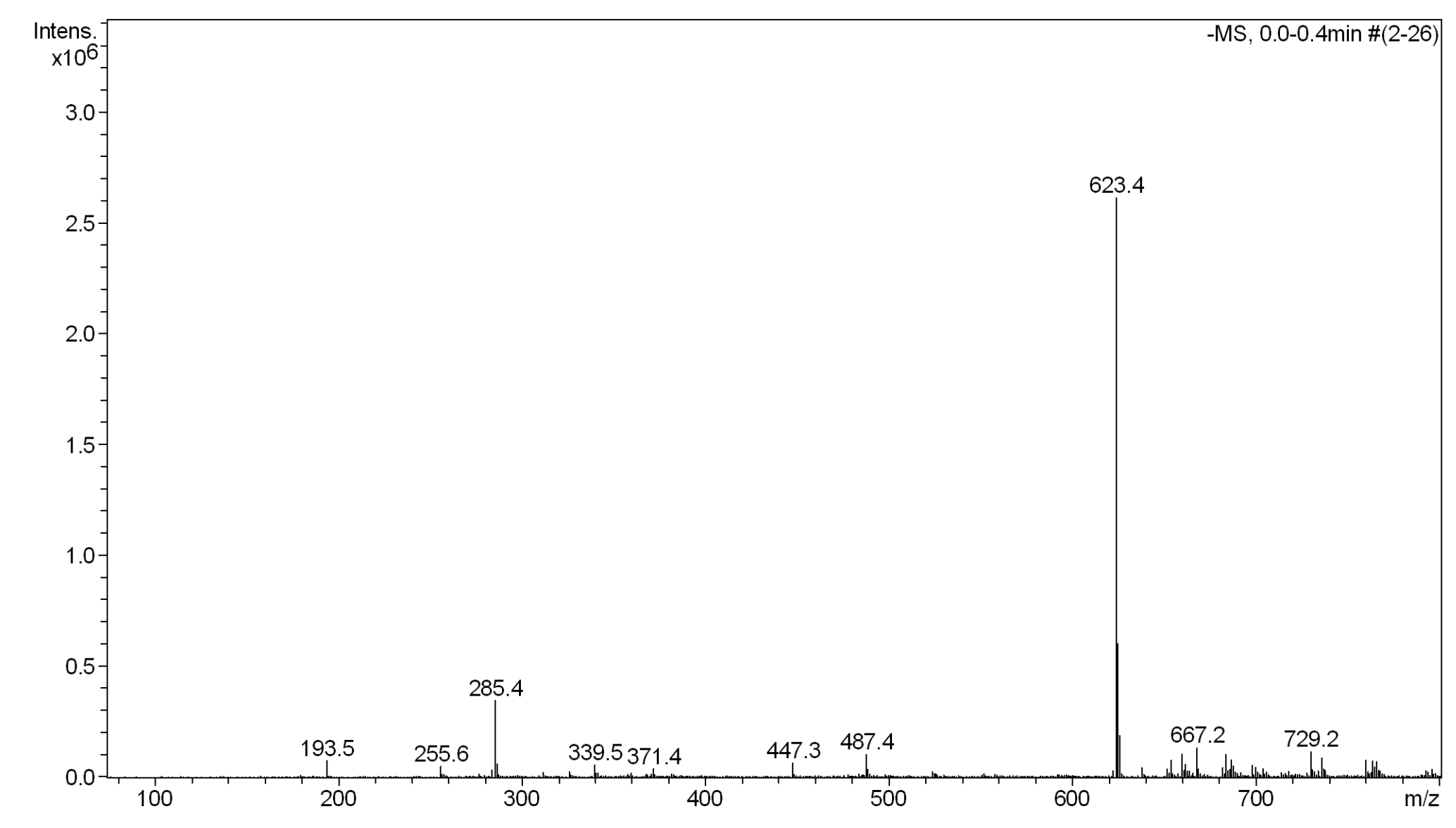


# Figure S7. (-) ESI-MS of acteoside.


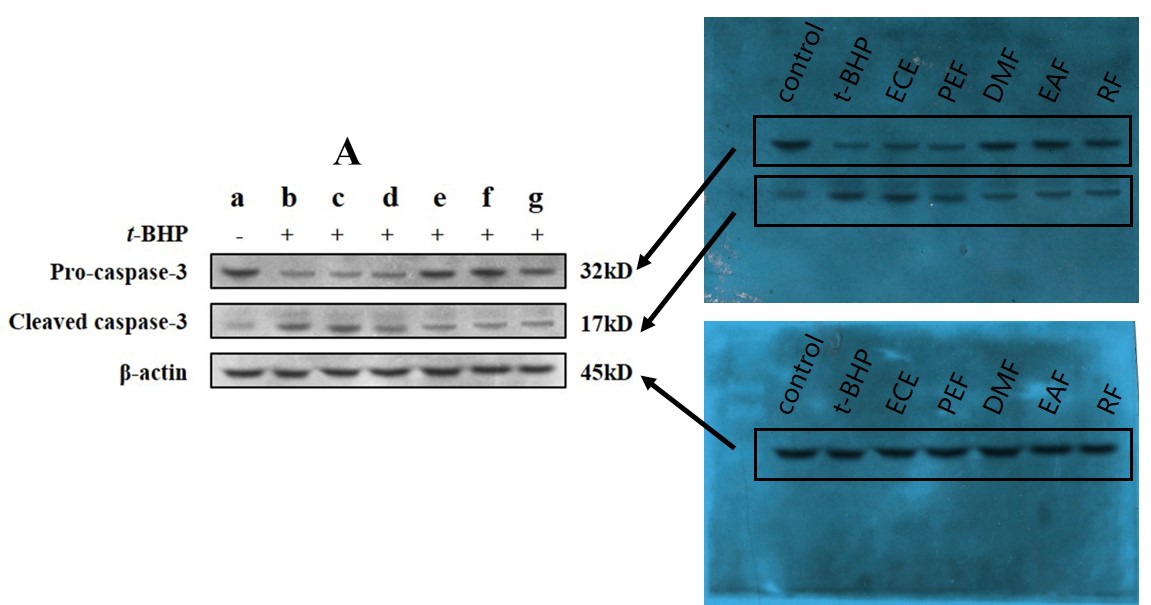


(1)


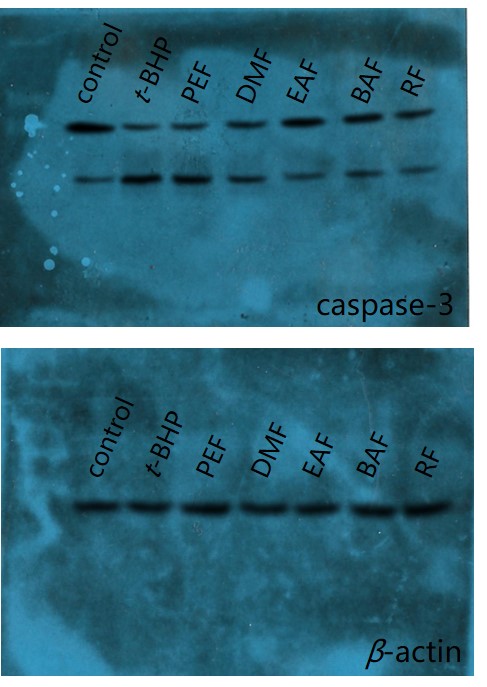


(2)


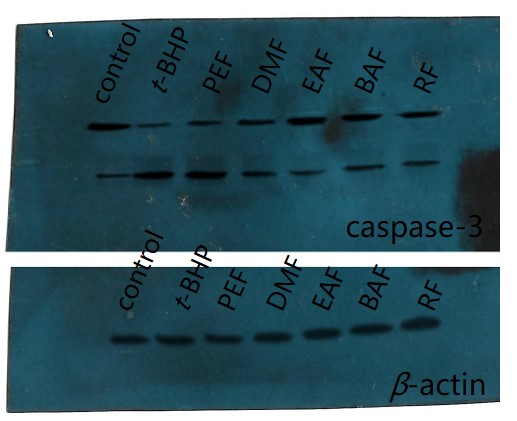


(3)

# Figure S8. Effect of ECE, PEF, DMF, EAF, BAF and RF on the expression of caspase-3 levels in HepG2 cells. Demonstration of original and final blots which were used in Figure 4(A). Protein levels of caspase-3 was detected by western blot with *β*-actin as an internal control. Triplicate analyses were performed and the results are presented in (1)-(3).


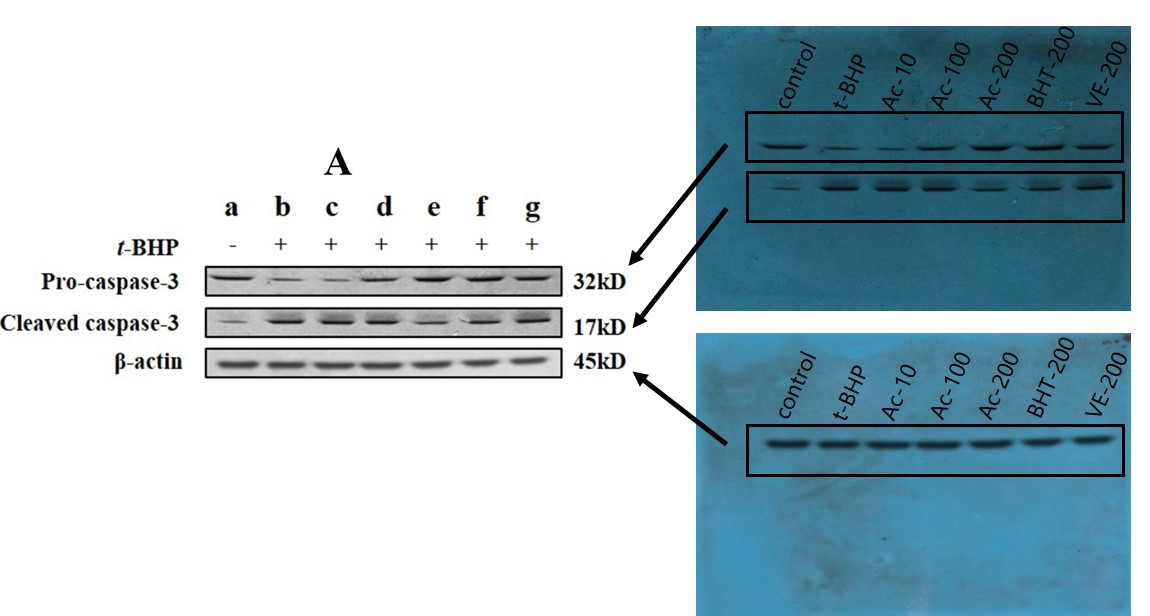


(1)


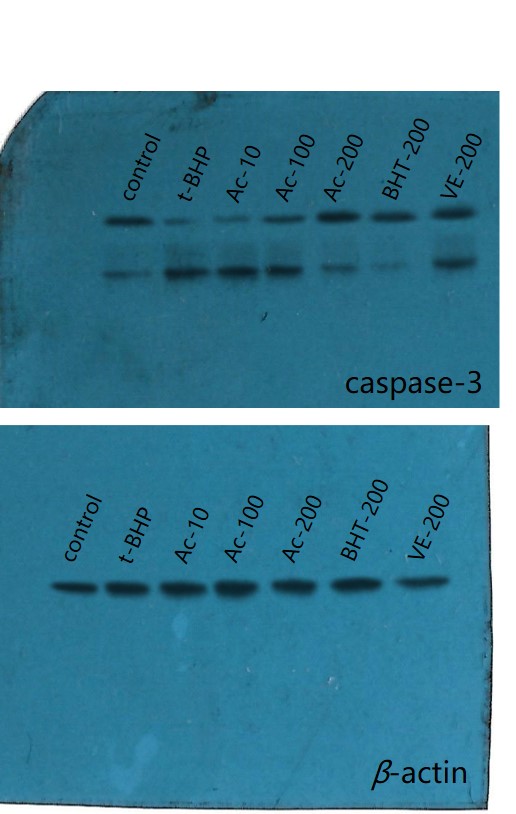


(2)


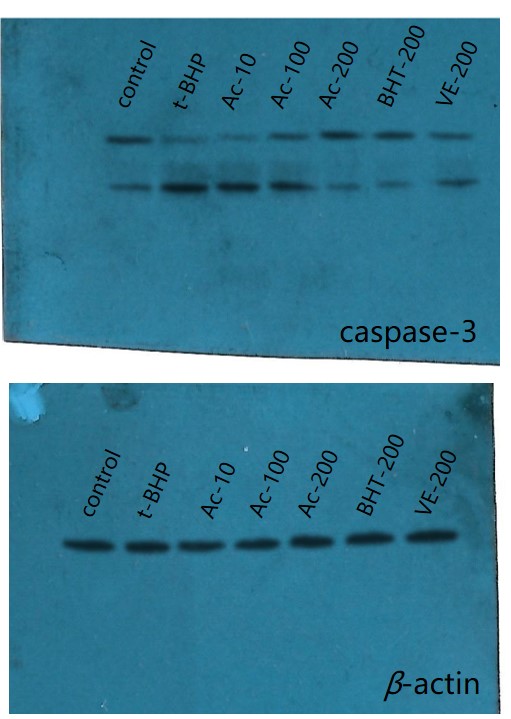


(3)

# Figure S9. Effect of Ac on the expression of caspase-3 levels in HepG2 cells. Demonstration of original and final blots which were used in Figure 6(A). Protein levels of caspase-3 was detected by western blot with *β*-actin as an internal control. Triplicate analyses were performed and the results are presented in (1)-(3).

# Table S1. Effect of t-BHP on HepG2 cell viability.

| *t*-BHP mmol/L | OD | Cell Viability % |
| --- | --- | --- |
| 0.0 | 0.581 ± 0.047 | 100.0 ± 8.2 |
| 0.2 | 0.567 ± 0.016 | 97.5 ± 2.8 |
| 0.4 | 0.506 ± 0.021 | 87.0 ± 3.7 |
| 0.5 | 0.425 ± 0.011 | 73.2 ± 1.9 |
| 0.6 | 0.359 ± 0.019 | 61.8 ± 3.3 |
| 0.7 | 0.285 ± 0.006 | 49.0 ± 1.0 |
| 0.8 | 0.204 ± 0.008 | 35.2 ± 1.4 |
| 0.9 | 0.139 ± 0.013 | 24.0 ± 2.2 |
| 1.0 | 0.101 ± 0.018 | 17.4 ± 3.1 |
| 1.2 | 0.046 ± 0.002 | 8.0 ± 0.4 |

# Table S2. Cytotoxicity of ECE, PEF, DMF, EAF, BAF and RF on HepG2 cells.

|  | OD | Cell Viability % | OD | Cell Viability % | OD | Cell Viability % | OD | Cell Viability % |
| --- | --- | --- | --- | --- | --- | --- | --- | --- |
| concentration μg/mL | 0 | | 10 | | 20 | | 30 | |
| control | 0.973 ± 0.029 | 100.0 ± 3.0 | - | - | - | - | - | - |
| ECE | - | - | 1.001 ± 0.053 | 103.0 ± 5.5 | 0.985 ± 0.039 | 101.2 ± 4.0 | 0.963 ± 0.042 | 99.0 ± 4.3 |
| PEF | - | - | 0.973 ± 0.023 | 100.1 ± 2.4 | 0.975 ± 0.050 | 100.2 ± 5.2 | 0.967 ± 0.020 | 99.4 ± 2.0 |
| DMF | - | - | 1.005 ± 0.059 | 103.3 ± 6.0 | 1.009 ± 0.054 | 103.7 ± 5.5 | 0.984 ± 0.057 | 101.2 ± 5.8 |
| EAF | - | - | 0.971 ± 0.034 | 99.8 ± 3.5 | 1.001 ± 0.053 | 102.9 ± 5.5 | 0.991 ± 0.065 | 101.9 ± 6.7 |
| BAF | - | - | 1.000 ± 0.043 | 102.8 ± 4.4 | 1.004 ± 0.057 | 103.3 ± 5.9 | 0.965 ± 0.020 | 99.3 ± 2.1 |
| RF | - | - | 1.009 ± 0.040 | 103.7 ± 4.1 | 0.984 ± 0.057 | 101.2 ± 5.8 | 1.001 ± 0.049 | 102.9 ± 5.0 |

# Table S3. The protective effect of ECE, PEF, DMF, EAF, BAF and RF on t-BHP -induced oxidative damage.

|  | OD | Cell Viability % | OD | Cell Viability % | OD | Cell Viability % | OD | Cell Viability % |
| --- | --- | --- | --- | --- | --- | --- | --- | --- |
| concentration | 0 | | 10 | | 20 | | 30 | |
| control | 0.534 ± 0.032 | 100.0 ± 5.9 | - | - | - | - | - | - |
| *t*-BHP | 0.255 ± 0.019 | 47.7 ± 3.5 | - | - | - | - | - | - |
| *t*-BHP + ECE | - | - | 0.266 ± 0.010 | 49.8 ± 1.9 | 0.303 ± 0.012 | 56.8 ± 2.2 | 0.344 ± 0.006 | 64.4 ± 1.1 |
| *t*-BHP + PEF | - | - | 0.248 ± 0.022 | 46.4 ± 4.1 | 0.260 ± 0.011 | 48.6 ± 2.1 | 0.270 ± 0.011 | 50.5 ± 2.1 |
| *t*-BHP + DMF | - | - | 0.254 ± 0.020 | 47.6 ± 3.8 | 0.261 ± 0.020 | 48.9 ± 3.8 | 0.276 ± 0.020 | 51.8 ± 3.8 |
| *t*-BHP + EAF | - | - | 0.290 ± 0.022 | 54.2 ± 4.0 | 0.321 ± 0.018 | 60.2 ± 3.5 | 0.423 ± 0.031 | 79.3 ± 5.9 |
| *t*-BHP + BAF | - | - | 0.277 ± 0.027 | 51.8 ± 5.0 | 0.309 ± 0.022 | 57.8 ± 4.0 | 0.374 ± 0.012 | 70.0 ± 2.3 |
| *t*-BHP + RF | - | - | 0.279 ± 0.016 | 52.3 ± 3.0 | 0.302 ± 0.021 | 56.6 ± 3.9 | 0.326 ± 0.022 | 61.0 ± 4.1 |

# Table S4. Cytotoxicity of Ac on HepG2 cells.

| Ac μmol/L | OD | Cell Viability % |
| --- | --- | --- |
| 0 | 0.973 ± 0.029 | 100.0 ± 3.0 |
| 1 | 0.975 ± 0.050 | 100.2 ± 5.2 |
| 10 | 0.967 ± 0.020 | 99.4 ± 2.0 |
| 100 | 1.005 ± 0.059 | 103.3 ± 6.0 |
| 200 | 1.009 ± 0.054 | 103.7 ± 5.5 |
| 300 | 0.984 ± 0.057 | 101.2 ± 5.8 |
| 400 | 1.000 ± 0.043 | 102.8 ± 4.4 |
| 600 | 0.951 ± 0.079 | 97.7 ± 8.1 |
| 1000 | 0.817 ± 0.083 | 84.0 ± 8.6 |

# Table S5. The protective effect of Ac on t-BHP -induced oxidative damage.

| Samples | OD | Cell Viability % |
| --- | --- | --- |
| control | 0.655 ± 0.052 | 100.0 ± 8.0 |
| *t*-BHP | 0.320 ± 0.024 | 48.8 ± 3.6 |
| t-BHP + Ac (1 μmol/L) | 0.335 ± 0.037 | 51.0 ± 5.6 |
| t-BHP + Ac (10 μmol/L) | 0.468 ± 0.056 | 71.4 ± 8.5 |
| t-BHP + Ac (100 μmol/L) | 0.549 ± 0.081 | 83.7 ± 12.4 |
| t-BHP + Ac (200 μmol/L) | 0.626 ± 0.036 | 95.5 ± 5.5 |
| t-BHP + Ac (300 μmol/L) | 0.590 ± 0.016 | 90.0 ± 2.5 |
| t-BHP + Ac (400 μmol/L) | 0.518 ± 0.013 | 79.0 ± 4.1 |
| t-BHP + BHT (200 μmol/L) | 0.622 ± 0.056 | 94.8 ± 8.5 |
| t-BHP + VE (200 μmol/L) | 0.526 ± 0.058 | 80.3 ± 8.9 |

# Table S6. Effect of ECE, PEF, DMF, EAF, BAF and RF on HepG2 intracellular ROS.

| Samples | A.U. | Intracellular ROS production % |
| --- | --- | --- |
| control | 5676.7 ± 469.1 | 100.0 ± 8.3 |
| *t*-BHP | 10218.0 ± 515.2 | 180.0 ± 9.1 |
| *t*-BHP + ECE | 8804.4 ± 253.4 | 155.1 ± 4.5 |
| *t*-BHP + PEF | 9610.7 ± 380.8 | 169.3 ± 6.7 |
| *t*-BHP + DMF | 9593.3 ± 891.1 | 169.0 ± 15.7 |
| *t*-BHP + EAF | 7657.7 ± 647.3 | 134.9 ± 11.4 |
| *t*-BHP + BAF | 7680.3 ± 360.7 | 135.3 ± 6.4 |
| *t*-BHP + RF | 8872.7 ± 239.7 | 156.3 ± 4.2 |

# Table S7. Effects of ECE, PEF, DMF, EAF, BAF and RF on LDH level.

| Samples | OD | lactate dehydrogenase activity U/gprot |
| --- | --- | --- |
| control | 0.261 ± 0.023 | 95.8 ± 11.2 |
| *t*-BHP | 0.383 ± 0.021 | 257.4 ± 16.4 |
| *t*-BHP + ECE | 0.346 ± 0.004 | 209.9 ± 8.1 |
| *t*-BHP + PEF | 0.378 ± 0.017 | 243.3 ± 11.8 |
| *t*-BHP + DMF | 0.371 ± 0.005 | 238.2 ± 15.5 |
| *t*-BHP + EAF | 0.306 ± 0.006 | 171.2 ± 18.8 |
| *t*-BHP + BAF | 0.336 ± 0.005 | 202.0 ± 18.6 |
| *t*-BHP + RF | 0.347 ± 0.003 | 208.8 ± 11.4 |

# Table S8. Effects of ECE, PEF, DMF, EAF, BAF and RF on MDA level.

| Samples | OD | Malondialdehyde nmol/mgprot |
| --- | --- | --- |
| control | 0.362 ± 0.084 | 11.4 ± 2.5 |
| *t*-BHP | 0.477 ± 0.024 | 24.9 ± 1.2 |
| *t*-BHP + ECE | 0.465 ± 0.073 | 17.6 ± 2.8 |
| *t*-BHP + PEF | 0.412 ± 0.041 | 22.1 ± 4.1 |
| *t*-BHP + DMF | 0.483 ± 0.120 | 22.4 ± 1.5 |
| *t*-BHP + EAF | 0.431 ± 0.013 | 15.9 ± 3.1 |
| *t*-BHP + BAF | 0.377 ± 0.020 | 17.0 ± 1.8 |
| *t*-BHP + RF | 0.454 ± 0.090 | 19.0 ± 2.6 |

# Table S9. Effects of ECE, PEF, DMF, EAF, BAF and RF on GSH level.

| Samples | OD | Glutathione μmol/gprot |
| --- | --- | --- |
| control | 0.146 ± 0.016 | 36.5 ± 5.4 |
| *t*-BHP | 0.096 ± 0.004 | 12.1 ± 5.3 |
| *t*-BHP + ECE | 0.122 ± 0.014 | 23.8 ± 4.1 |
| *t*-BHP + PEF | 0.098 ± 0.004 | 10.9 ± 2.6 |
| *t*-BHP + DMF | 0.112 ± 0.013 | 16.1 ± 4.1 |
| *t*-BHP + EAF | 0.138 ± 0.018 | 29.3 ± 1.3 |
| *t*-BHP + BAF | 0.134 ± 0.012 | 25.1 ± 1.1 |
| *t*-BHP + RF | 0.122 ± 0.014 | 27.3 ± 4.0 |

# Table S10. Effects of ECE, PEF, DMF, EAF, BAF and RF on SOD activity.

| Samples | OD | Superoxide dismutase activity U/mgprot |
| --- | --- | --- |
| control | 0.140 ± 0.053 | 188.3 ± 7.1 |
| *t*-BHP | 0.361 ± 0.051 | 101.3 ± 5.1 |
| *t*-BHP + ECE | 0.310 ± 0.028 | 130.2 ± 6.1 |
| *t*-BHP + PEF | 0.373 ± 0.048 | 102.7 ± 14.9 |
| *t*-BHP + DMF | 0.358 ± 0.053 | 121.0 ± 2.0 |
| *t*-BHP + EAF | 0.269 ± 0.083 | 154.4 ± 8.3 |
| *t*-BHP + BAF | 0.294 ± 0.039 | 140.3 ± 4.6 |
| *t*-BHP + RF | 0.238 ± 0.053 | 135.9 ± 6.2 |

# Table S11. Effects of ECE, PEF, DMF, EAF, BAF and RF on CAT activity.

| Samples | OD | Catalase activity U/mgprot |
| --- | --- | --- |
| control | 0.367 ± 0.039 | 21.1 ± 0.8 |
| *t*-BHP | 0.474 ± 0.020 | 11.9 ± 0.5 |
| *t*-BHP + ECE | 0.462 ± 0.017 | 13.9 ± 0.7 |
| *t*-BHP + PEF | 0.471 ± 0.014 | 12.1 ± 0.7 |
| *t*-BHP + DMF | 0.475 ± 0.013 | 13.2 ± 0.6 |
| *t*-BHP + EAF | 0.445 ± 0.028 | 14.8 ± 0.3 |
| *t*-BHP + BAF | 0.441 ± 0.031 | 14.3 ± 0.7 |
| *t*-BHP + RF | 0.448 ± 0.027 | 13.9 ± 0.7 |

# Table S12. Effect of Ac on HepG2 intracellular ROS.

| Samples | A.U. | Intracellular ROS production % |
| --- | --- | --- |
| control | 7179.3 ± 539.3 | 100.0 ± 7.5 |
| *t*-BHP | 14158.5 ± 1078.5 | 197.2 ± 15.0 |
| *t*-BHP + Ac (10 μmol/L) | 13012.0 ± 390.1 | 181.2 ± 5.4 |
| *t*-BHP + Ac (50 μmol/L) | 11157.3 ± 725.0 | 155.4 ± 10.1 |
| *t*-BHP + Ac (100 μmol/L) | 8113.0 ± 359.7 | 113.0 ± 5.0 |
| *t*-BHP + Ac (200 μmol/L) | 9235.3 ± 1323.8 | 128.6 ± 18.4 |
| *t*-BHP + BHT (200 μmol/L) | 9549.0 ± 1050.6 | 133.0 ± 14.6 |
| *t*-BHP + VE (200 μmol/L) | 10329.3 ± 999.2 | 143.9 ± 13.9 |

# Table S13. Effects of Ac on LDH level.

| Samples | OD | lactate dehydrogenase activity U/gprot |
| --- | --- | --- |
| control | 0.261 ± 0.023 | 95.8 ± 11.2 |
| *t*-BHP | 0.383 ± 0.021 | 257.4 ± 16.4 |
| *t*-BHP + Ac (10 μmol/L) | 0.347 ± 0.022 | 210.0 ± 13.2 |
| *t*-BHP + Ac (50 μmol/L) | 0.341 ± 0.019 | 205.8 ± 10.3 |
| *t*-BHP + Ac (100 μmol/L) | 0.296 ± 0.022 | 160.0 ± 13.1 |
| *t*-BHP + Ac (200 μmol/L) | 0.288 ± 0.021 | 148.4 ± 9.3 |
| *t*-BHP + BHT (200 μmol/L) | 0.289 ± 0.020 | 145.0 ± 8.5 |
| *t*-BHP + VE (200 μmol/L) | 0.317 ± 0.022 | 174.8 ± 13.0 |

# Table S14. Effects of Ac on MDA level.

| Samples | OD | Malondialdehyde nmol/mgprot |
| --- | --- | --- |
| control | 0.466 ± 0.042 | 16.5 ± 2.0 |
| *t*-BHP | 0.572 ± 0.086 | 30.9 ± 1.1 |
| *t*-BHP + Ac (10 μmol/L) | 0.745 ± 0.167 | 28.0 ± 2.4 |
| *t*-BHP + Ac (50 μmol/L) | 0.713 ± 0.188 | 26.7 ± 2.6 |
| *t*-BHP + Ac (100 μmol/L) | 0.519 ± 0.069 | 20.4 ± 4.5 |
| *t*-BHP + Ac (200 μmol/L) | 0.450 ± 0.146 | 18.6 ± 2.5 |
| *t*-BHP + BHT (200 μmol/L) | 0.453 ± 0.053 | 19.6 ± 2.6 |
| *t*-BHP + VE (200 μmol/L) | 0.475 ± 0.051 | 19.4 ± 4.6 |

# Table S15. Effects of Ac on GSH level.

| Samples | OD | Glutathione μmol/gprot |
| --- | --- | --- |
| control | 0.160 ± 0.010 | 43.0 ± 2.0 |
| *t*-BHP | 0.089 ± 0.002 | 10.3 ± 3.4 |
| *t*-BHP + Ac (10 μmol/L) | 0.106 ± 0.009 | 18.6 ± 5.5 |
| *t*-BHP + Ac (50 μmol/L) | 0.107 ± 0.014 | 21.3 ± 4.3 |
| *t*-BHP + Ac (100 μmol/L) | 0.115 ± 0.008 | 33.8 ± 1.4 |
| *t*-BHP + Ac (200 μmol/L) | 0.148 ± 0.011 | 42.0 ± 5.1 |
| *t*-BHP + BHT (200 μmol/L) | 0.138 ± 0.029 | 36.8 ± 1.9 |
| *t*-BHP + VE (200 μmol/L) | 0.129 ± 0.009 | 30.6 ± 2.6 |

# Table S16. Effects of Ac on SOD activity.

| Samples | OD | Superoxide dismutase activity U/mgprot |
| --- | --- | --- |
| control | 0.140 ± 0.053 | 188.3 ± 7.1 |
| *t*-BHP | 0.361 ± 0.051 | 101.3 ± 5.1 |
| *t*-BHP + Ac (10 μmol/L) | 0.267 ± 0.078 | 133.6 ± 7.8 |
| *t*-BHP + Ac (50 μmol/L) | 0.302 ± 0.077 | 149.8 ± 5.1 |
| *t*-BHP + Ac (100 μmol/L) | 0.246 ± 0.072 | 168.3 ± 6.2 |
| *t*-BHP + Ac (200 μmol/L) | 0.200 ± 0.090 | 173.6 ± 4.8 |
| *t*-BHP + BHT (200 μmol/L) | 0.249 ± 0.079 | 170.2 ± 6.8 |
| *t*-BHP + VE (200 μmol/L) | 0.229 ± 0.087 | 160.9 ± 8.6 |

# Table S16. Effects of Ac on CAT activity.

| Samples | OD | Catalase activity U/mgprot |
| --- | --- | --- |
| control | 0.367 ± 0.039 | 21.1 ± 0.8 |
| *t*-BHP | 0.474 ± 0.020 | 11.9 ± 0.5 |
| *t*-BHP + Ac (10 μmol/L) | 0.460 ± 0.031 | 13.3 ± 0.7 |
| *t*-BHP + Ac (50 μmol/L) | 0.456 ± 0.021 | 14.3 ± 0.5 |
| *t*-BHP + Ac (100 μmol/L) | 0.396 ± 0.037 | 17.8 ± 0.6 |
| *t*-BHP + Ac (200 μmol/L) | 0.366 ± 0.031 | 18.5 ± 0.5 |
| *t*-BHP + BHT (200 μmol/L) | 0.374 ± 0.026 | 18.4 ± 0.5 |
| *t*-BHP + VE (200 μmol/L) | 0.412 ± 0.037 | 17.4 ± 0.6 |

# Table S17. Data of Figure 4 analyzed by ImageJ software.

|  | *β*-actin | | | pro-caspase-3 | | | cleaved caspase-3 | | |
| --- | --- | --- | --- | --- | --- | --- | --- | --- | --- |
|  | integrated density | ratio | | integrated density | ratio | | integrated density | ratio | |
| control | 293699 ± 26333 | | - | 190872 ± 10492 | | 0.66 ± 0.09 | 86337 ± 25385 | | 0.30 ± 0.11 |
| *t*-BHP | 250478 ± 10353 | | - | 92131 ± 7278 | | 0.37 ± 0.04 | 173553 ± 3860 | | 0.69 ± 0.04 |
| *t*-BHP + PEF | 251987 ± 11171 | | - | 86415 ± 4074 | | 0.34 ± 0.02 | 173035 ± 9523 | | 0.69 ± 0.05 |
| *t*-BHP + DMF | 273986 ± 10755 | | - | 112302 ± 6420 | | 0.41 ± 0.03 | 140275 ± 14546 | | 0.51 ± 0.06 |
| *t*-BHP + EAF | 266022 ± 12515 | | - | 162479 ± 6998 | | 0.61 ± 0.01 | 102151 ± 8829 | | 0.39 ± 0.05 |
| *t*-BHP + BAF | 276669 ± 11435 | | - | 160529 ± 10195 | | 0.58 ± 0.02 | 101154 ± 3332 | | 0.37 ± 0.03 |
| *t*-BHP + RF | 252199 ± 9497 | | - | 123817 ± 8512 | | 0.49 ± 0.04 | 121503 ± 5760 | | 0.48 ± 0.03 |

# Table S18. Data of Figure 6 analyzed by ImageJ software.

|  | *β*-actin | | | pro-caspase-3 | | | cleaved caspase-3 | | |
| --- | --- | --- | --- | --- | --- | --- | --- | --- | --- |
|  | integrated density | ratio | | integrated density | ratio | | integrated density | ratio | |
| control | 420285 ± 4815 | | - | 480795 ± 38871 | | 1.14 ± 0.09 | 132186 ± 24456 | | 0.31 ± 0.06 |
| *t*-BHP | 472090 ± 9918 | | - | 162666 ± 48254 | | 0.35 ± 0.10 | 379327 ± 64198 | | 0.80 ± 0.15 |
| *t*-BHP + Ac (10 μmol/L) | 513995 ± 5923 | | - | 129212 ± 26146 | | 0.25 ± 0.05 | 365883 ± 49749 | | 0.71 ± 0.10 |
| *t*-BHP + Ac (100 μmol/L) | 530340 ± 9390 | | - | 342195 ± 33679 | | 0.65 ± 0.07 | 327823 ± 51278 | | 0.62 ± 0.11 |
| *t*-BHP + Ac (200 μmol/L) | 491811 ± 10825 | | - | 444668 ± 36407 | | 0.91 ± 0.09 | 224146 ± 28317 | | 0.46 ± 0.05 |
| *t*-BHP + BHT (200 μmol/L) | 427247 ± 5309 | | - | 462519 ± 50251 | | 1.08 ± 0.13 | 278314 ± 48521 | | 0.65 ± 0.12 |
| *t*-BHP + VE (200 μmol/L) | 426602 ± 5398 | | - | 353815 ± 50264 | | 0.83 ± 0.12 | 330679 ± 28360 | | 0.78 ± 0.07 |
